# Highly Multiplexed Imaging Uncovers Changes in Compositional Noise within Assembling Focal Adhesions

**DOI:** 10.1371/journal.pone.0160591

**Published:** 2016-08-12

**Authors:** Jana Harizanova, Yessica Fermin, Rahuman S. Malik-Sheriff, Jakob Wieczorek, Katja Ickstadt, Hernán E. Grecco, Eli Zamir

**Affiliations:** 1 Department of Systemic Cell Biology, Max Planck Institute of Molecular, Physiology, Dortmund, Germany; 2 Faculty of Statistics, TU Dortmund University, Dortmund, Germany; Istituto per la Ricerca e la Cura del Cancro di Candiolo, ITALY

## Abstract

Integrin adhesome proteins bind each other in alternative manners, forming within the cell diverse cell-matrix adhesion sites with distinct properties. An intriguing question is how such modular assembly of adhesion sites is achieved correctly solely by self-organization of their components. Here we address this question using high-throughput multiplexed imaging of eight proteins and two phosphorylation sites in a large number of single focal adhesions. We found that during the assembly of focal adhesions the variances of protein densities decrease while the correlations between them increase, suggesting reduction in the noise levels within these structures. These changes correlate independently with the area and internal density of focal adhesions, but not with their age or shape. Artificial neural network analysis indicates that a joint consideration of multiple components improves the predictability of paxillin and zyxin levels in internally dense focal adhesions. This suggests that paxillin and zyxin densities in focal adhesions are fine-tuned by integrating the levels of multiple other components, thus averaging-out stochastic fluctuations. Based on these results we propose that increase in internal protein densities facilitates noise suppression in focal adhesions, while noise suppression enables their stable growth and further density increase—hence forming a feedback loop giving rise to a quality-controlled assembly.

## Introduction

Cell-matrix adhesion sites are heterogeneous structures that assemble by a rapid self-organization of their components, collectively called the integrin adhesome [1–5]. Different types of these sites, including focal complexes, focal adhesions and fibrillar adhesions, have distinct molecular compositions underlying their specific functions [3, 4]. Additionally, adhesion sites of the same type can have different molecular compositions in response to different local cues [6–9]. The molecular diversity of cell-matrix adhesion sites is enabled by the alternative manners in which integrin adhesome proteins can bind each other [1, 5]. However, this also implies that during the assembly and maintenance of focal adhesions noise can arise from the stochastic realizations of alternative binding options between recruited and recruiting proteins. The noise in the level of a protein in a focal adhesion is the stochastic deviation of this level from the exact realization of all the binding probabilities in the system. Plausibly, such exact realizations reflect the evolutionarily tuned optimal levels of each component for the function of focal adhesions. As the assembly of focal adhesions is a multistep process, stochastic deviation from optimal molecular content might accumulate along the steps and lead to the formation of aberrant structures. Therefore, an intriguing question is how focal adhesions assemble correctly solely by self-organization of their components, in spite of their alternative binding possibilities. Here we address this question by assessing changes in noise levels in the molecular composition of assembling focal adhesions.

To monitor the molecular composition of focal adhesions, their components should be co-imaged at a high spatial resolution [9, 10]. However, due to the fundamental trade-offs, the number of proteins that can be co-imaged in the same sample at sufficient spatial resolution is limited [11]. Mass-spectrometry imaging enables monitoring high number of proteins and phosphorylation states [12, 13], but currently lacks a sufficient spatial resolution for analyzing individual adhesion sites [14]. Fluorescence microscopy provides sufficient spatial resolution, however the number of components that can be co-imaged is confined by the spectral overlap between fluorophores [9, 11]. Cyclic immunofluorescence (CycIF; also termed toponome imaging) bypasses this limitation by subjecting fixed cells to cycles of immunolabeling, imaging and bleaching (or other labeling inactivation methods), hence enabling multiplex imaging using the same fluorophore [11, 15–22]. Here we implemented high-throughput CycIF to image ten different components in thousands of individual focal adhesions. Based on changes in the variances of the densities of these components and in the correlations between them, we inferred changes in noise levels in focal adhesions. Under certain assumptions, a reduction in density variances accompanied with elevation in correlation strengths between them is indicative of noise reduction, and vice versa. Accordingly, we infer that during the assembly of focal adhesions the noise in their molecular content is reduced. This noise reduction correlates with the area and internal density of focal adhesions, but neither with their age nor their eccentricity. Using artificial neural networks analysis, we found that the levels of paxillin and zyxin in internally dense focal adhesions are better predicted if the densities of multiple other components are jointly considered. This suggests that at high internal density, the levels of paxillin and zyxin gain robustness to stochastic noise by integrating cues from multiple components in the focal adhesions. Based on these results, we suggest a model in which a positive feedback between internal density, noise suppression and growth of focal adhesions gives rise to compositional quality control in the assembly of these structures.

## Materials and Methods

### Cells and reagents

REF52 YFP-paxillin stable cell line (kindly provided by Benjamin Geiger and Joachim Spatz) were cultured in DMEM (PAN Biotech, Aidenbach, Germany) supplemented with 1% glucose, 10% fetal calf serum, 1% nonessential amino acids and 1% L-glutamine and maintained at 37°Cand 5% CO_2_. The primary antibodies included anti-vinculin mouse IgG1, anti-zyxin rabbit IgG, anti-*α*-actinin mouse IgM (V9264, Z4751, A5044; Sigma-Aldrich Chemie GmbH, Taufkirchen, Germany), anti-paxillin-pY118 rabbit IgG, anti-FAK-pY397 rabbit IgG (44-722G, 44-624G; Invitrogen GmbH, Karlsruhe, Germany), anti-VASP rabbit IgG (3132, Cell Signaling Technology, Frankfurt, Germany), Alexa Fluor 555 conjugated anti-FAK mouse IgG1 (clone 4.47, 16-234, Merck Millipore, Darmstadt, Germany), TRITC conjugated anti-paxillin mouse IgG1 and anti-Hic-5 mouse IgG1 (610055, 611164; BD Transduction Laboratories). F-actin was labeled with Alexa Fluor 350 Phalloidin (A-22281, Invitrogen GmbH). A secondary antibody for *α*-actinin staining was Alexa Fluor 350 goat anti-mouse IgM (A-31552, Invitrogen GmbH). The other unconjugated primary antibodies were pre-labeled with Zenon kit (Z-25041, Z-25306, Z-25006, Z-25308, Z-25008, Invitrogen GmbH) according to manufacturer protocol.

### Microscopy

REF52 YFP-paxillin cells were seeded at 70% confluence in 8-well Lab-Tek chamber slide (Nunc, Thermo Fisher Scientific, MA, USA), grown for 12-14 hours, washed with pre-warmed PBS and incubated in imaging medium (Pan Biotech, Aidenbach, Germany) supplemented with 4.5 g/l glucose, 25 mM HEPES, 0.5 g/l NaHCO_3_ and 10% fetal calf serum. The chamber slide was immobilized on the stage of an inverted wide-field microscope (Cell-R, Olympus, Hamburg, Germany) equipped with CCD camera (Orca R2, Hamamatsu Photonics, Hamamatsu, Japan), motorized stage, live-cell imaging chamber and controlled by an in-house software based on LabView (National Instruments, Austin, TX, USA). In order to maintain precise *z*-position of the objective, the CCD Laser Displacement Sensor (LK-3100, Keyence, Neu-Isenburg, Germany) was mounted on the microscope objective revolver. A set of field of views was selected and the corresponding microscope stage coordinates were stored. Time-lapse image sequences of YFP-paxillin were acquired for the stored positions using a dry 40x/.90 NA objective (Olympus, Japan) with an interval of 3 minutes between frames for a period of about 30 minutes, till fixation for CycIF. Following pre-CycIF, the cells were washed with pre-warmed PBS, fixed with 3% paraformaldehyde in PBS for 10 minutes, washed with PBS and permeabilized for 5 minutes with 0.2% Triton X-100 in PBS. CycIF imaging was applied here for labeling intracellular proteins, which requires cell permeabilization and longer incubations during immunolabling. To avoid an excessively long duration of the whole CycIF procedure, in each CycIF cycle two or three components where labeled and imaged, rather than one (S1 Table). Each CycIF cycle consists of labeling, imaging and bleaching steps. In the labeling step, the primary antibodies assigned for the given CycIF cycle were pre-lableled when needed, mixed and applied on the cells for 1 hour. The cells were then washed 3 times for 5 minutes each with washing buffer (0.02% Triton X-100 in PBS), incubated with a secondary antibody for 1 hour when *α*-actinin antibody was used, washed 3 times for 5 minutes each with washing buffer and kept in PBS for imaging. In the imaging step, images were taken for each stored position with excitation, beam splitter and emission filters of 350/50, 409, 447/60 (for Alexa 350 and Pacific Blue), 495/10, 505, 542/27 (for YFP), 545/20, 565, 597.5/55 (for Alexa 555 or Alexa 568) and 643/20, 660, 700/75 (for Alexa 647). At each CycIF cycle, an image of the YFP-paxillin localization was also acquired to provide a common basis for image registration and segmentation of adhesion sites. In the bleaching step, each position was exposed to excitation wavelengths corresponding to the labeling fluorophores, beside YFP, until no fluorescent signal was detected. To assess if potential steric hindrance between the antibodies affects the results, equivalent cell populations were subjected to two opposite orders of labeling cycles (S1 Table). Overall, 6 datasets of focal adhesions were acquired from 3 repeats (R1, R2 and R3) of the two labeling orders (O1 and O2), denoted accordingly R1O1, R1O2, R2O1, R2O2, R3O1 and R3O2 (S1–S3 Figs).

### Image analysis

Images were registered using single-step discrete Fourier transform algorithm [23] and background-subtracted using high-pass filtration. Adhesion sites were segmented based on the YFP-paxillin images using the watershed algorithm [3] implemented in Matlab (MathWorks, Massachusetts, USA). Based on this segmentation, the levels of the labeled components in each adhesion site were quantified in their corresponding images while the area and eccentricity of these sites were derived from the YFP-paxillin image. Eccentricity was calculated as the ratio of the distance between the foci of the ellipse and its major axis length. The identity of the segmented adhesion sites as focal adhesions was verified based on their zyxin content [24, 25] and oval shape (S2 Fig). Noteworthy, the thickness of focal adhesions is rather independent of their area [26], therefore the fluorescence intensity of a labeled protein in focal adhesions reflects its density. To derive the dynamic history of each focal adhesion in the post-fixation images, the last four pre-fixation YFP-paxillin images were segmented as abovementioned and focal adhesions were tracked backward semi-automatically using a custom-made Matlab software. Focal adhesions in sequential images were automatically matched based on maximum overlap of their area, or shortest distance between their center of mass within a radius of 10 pixels if no area overlap was found. These matches were then inspected manually and corrected where needed. Focal adhesions that were absent in the post-fixation images were excluded from further analysis. The tracked focal adhesions were categorized primarily based on how early they appeared prior fixation: >12’, 12’, 9’, 6’ and 3’ -old. The >12’-old focal adhesions were further sub-categorized based on the change in their YFP-paxillin levels along these frames: AS (assembling) for those exhibiting an increase, ST (stationary) for those exhibiting no significant change and DS (disassembling) for those exhibiting a decrease in the total levels of YFP-paxillin. This sub-categorization was done according to the Pearson correlation coefficient between the total levels of YFP-paxillin and time, so that focal adhesions with values below -0.7, between -0.7 and 0.7 or above 0.7 were classified as DS, ST and AS, respectively.

### Data processing

The density of a given component in a given focal adhesion was calculated as the sum of the intensity in its corresponding pixels in the corresponding image divided by the number of these pixels. Then, the Box-Cox transformation [27] was applied such that *x*(*λ*_1_, *λ*_2_) = ((*y* + *λ*_2_)^*λ*1^ − 1)/*λ*_1_ if *λ*_1_ ≠ 0, *x*(*λ*_1_,*λ*_2_) = *log*(*y* + *λ*_2_) if *λ*_1_ = 0, where the optimal transformation parameter *λ*_1_ was calculated as the mean of individually determined transformation parameters for each labeled component within a single dataset using the maximum likelihood approach [27]. The transformed data were then normalized by subtracting the mean and dividing by the standard deviation and then outliers were removed from the obtained *z*-scores using Tukey’s approach [28]: *z*_*out*_ ∉ [*Q*_1_ − 2*IQR*; *Q*_3_ + 2*IQR*] where *Q*_1_ is the lower quartile, *Q*_3_ is the upper quartile and *IRQ* = *Q*_3_ − *Q*_1_. Where indicated, focal adhesions were categorized according to their area and eccentricity using Otsu’s multilevel thresholding [29] on combined parameter values from all 6 datasets. This yielded two threshold levels for each classifying parameter: 2.71 *μ*m^2^ and 6.17 *μ*m^2^ for area and 0.86 and 0.92 for eccentricity. To classify focal adhesions based on their internal density, the densities of each labeled component in focal adhesions, besides that of FAK-pY397 and paxillin-pY118, were normalized by subtracting their mean and dividing by their standard deviation per component per dataset, and then averaged per focal adhesion. Based on these mean normalized densities, focal adhesions were categorized using the above-mentioned Otsu’s method, which yielded the thresholds of -0.42 and 0.282.

### Simulations

The statistical properties of proteins in focal adhesions were derived from stochastic simulations (S1 Text and S4–S6 Figs). The number of recruited proteins for each focal adhesion was obtained from realizations of the binomial distribution to bind to a number of identical sites with a given probability. While enzymatic reactions and feedback loops are certainly present in focal adhesions, the number of possible networks and the size of the parameter space hinders the ability to explicitly consider them. Therefore we have followed another path in which these topological motifs regulate effective parameters: (i) higher diversity among focal adhesions was introduced by widening the distribution from which the number of binding sites was drawn, (ii) higher noise was introduced by widening the distribution from which the binding probability, related to the binding kinetics, was drawn. Thus, we simulated different variations of assembly models, including: (i) competitive and non-competitive recruitment, (ii) multi-step assembly, (iii) diversities generated from uniform, normal and Poisson distributions, (iv) different relations between the range of the diversity and its mean and (v) different stoichiometry between the recruited proteins. For all cases, the *r*^2^ and *CV* were calculated and used to validate the noise-inference approach (S1 Text).

### Inferring changes in diversity and noise levels

For each data set and focal adhesions category (e.g. focal adhesions within a given size range), the *CV* of the level of each component and the *r*^2^ between each pair combination, were calculated. The change in the *CV* between two compared focal adhesion categories (e.g. small versus big focal adhesions) was calculated first for each component as *log*(*CV*_2_/*CV*_1_). The change in *r*^2^ was calculated first for each pair of components as their $r_{2}^{2}-r_{1}^{2}$. Then, these values were averaged, first over all datasets having at least 5 focal adhesions in each compared group (S1 Fig) and next over all components, to give the Δ*log*(*CV*_1_,*CV*_2_) and $\Delta (r_{1}^{2},r_{2}^{2})$, respectively. If Δ*log*(*CV*_1_,*CV*_2_) is negative and $\Delta (r_{1}^{2},r_{2}^{2})$ is positive it was concluded that the noise is lower in category 2, while in the opposite case that noise is lower in category 1 (S1 Text). If the absolute value of either Δ*log*(*CV*_1_,*CV*_2_) or $\Delta (r_{1}^{2},r_{2}^{2})$ was smaller than the corresponding standard error of the mean then the sign was considered insignificant and therefore the particular inference as inconclusive.

### Inferring high-order relations

Three-layer perceptrons applying Levenberg-Marquardt back-propagation learning (Neural Networks Toolbox, Matlab 2010, Mathworks) were used to test if information about the densities of a given subset of labeled components (input components) is sufficient for predicting the density of another given labeled component (target component) in focal adhesions. The analysis included the following steps: in the first step, the input and target data for training and evaluating the artificial neural network were organized. A given component was considered as a target protein, while all possible combinations of the remaining 9 components were used to generate 511 separate inputs. In the second step, the network architecture was set to consist of three hidden layers with number of neurons in each layer equals the number of input components. In the third step, for each input-target combination, 500 independent sessions of training, validation and testing were performed, each session using the components densities of randomly sampled 40%, 30% and 30% focal adhesions, respectively. In the fourth step, the performance was evaluated based on the mean squared error between the target and predicted output after training using the complete dataset. This analysis was performed on each of the six datasets independently for evaluation of reproducibility. Random Forests analysis [30] was performed on the same input and target data used for the artificial neural networks analysis, using the randomForest package in R statistical system version 3.0.1, with 500 trees to grow and selecting all proteins in the respective input proteins combination as candidate at each node. Based on the artificial neural networks analysis results, for each target component, a directed acyclic graph (DAG) was constructed with 511 nodes corresponding to possible combinations of input components and edges between each two nodes that differ only due to the presence or absence of one component. The directionality of each edge was set to point toward the node that predicts significantly better the target protein, as determined using an F-test. The position of a node within the DAG was then scored as *L* = *L*1/(*L*1 + *L*2), where *L*1 and *L*2 are the number of edges to the closest single protein and null out-degree node respectively. Accordingly, nodes that have high *L* value (> 0.7) in at least 4 data sets (at least 2 from of each labeling order) were selected as indicating high-order relations. The construction of the graphs was done using Matlab and the graph analysis calculations were written in Python 2.7.9 using the package NetworkX 1.9.1.

## Results

### Multiplexed imaging of focal adhesions

In order to investigate the noise in the molecular composition of focal adhesions, it is required to co-image the levels of a large number of components in a large number of individual focal adhesions. We extended CycIF [15, 17] to enable multiplexed imaging of a large number of proteins within small intracellular structures (see Materials and Methods). For this the following aspects were addressed: (i) To facilitate the long incubation and washout durations required for immunofluorescence labeling of intracellular proteins, three or two components were labeled and imaged in each CycIF, hence reducing the total number of cycles. (ii) While CycIF overcomes limitations due to spectral overlap between fluorophores, it still requires specific immuno-labeling of each component. Indirect immuno-fluorescence would strongly limit the number of components that can be imaged by CycIF, since cross-talk between the secondary and primary antibodies has to be avoided. Direct immunofluorescence does not have this limitation, but unlike for many cell surface proteins, fluorescently conjugated primary antibodies for cytosolic adhesion site components are scarcely available. As a generic solution for fluorescently labeling of primary antibodies, we pre-complexed them with fluorescently labeled Fab fragments. (iii) To control for possible accessibility limitations of antibodies to their antigens within focal adhesions, two different labeling cycle orders were applied and compared.

**Fig 1:**
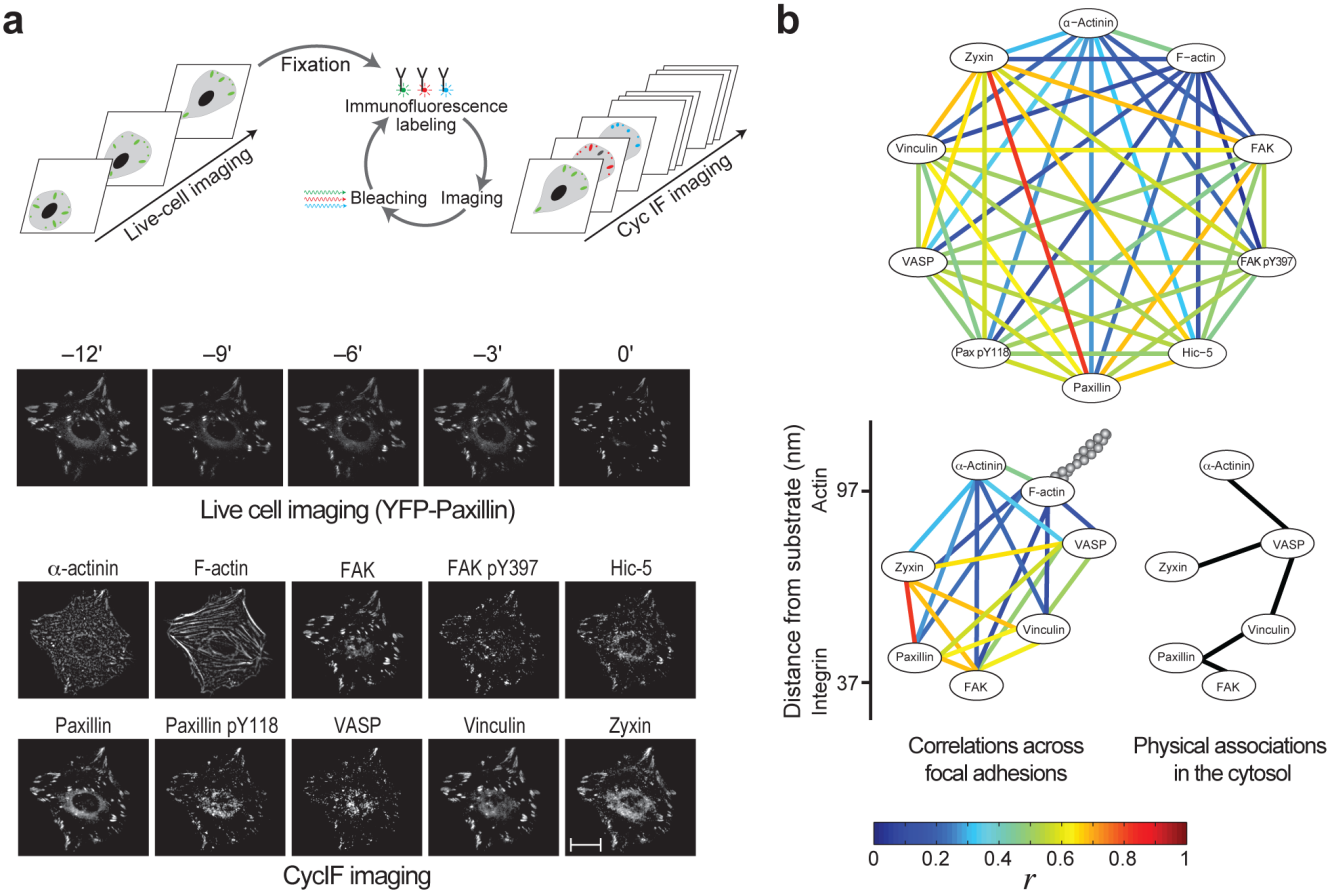
High-throughput CycIF imaging of cell-matrix adhesion sites. (a) Imaging procedure and an example of the images obtained for a cell. Scale bar, 10 *μ*m. (b) Top, the mean Pearson correlation coefficients (*r*, *n* = 6 datasets; see S2 Table) between the densities of the components. Bottom, superposition of these correlations with the reported vertical positions of the components across focal adhesions [26] (left) and a comparison with their reported physical associations in the cytosol [33] (right).

The above-mentioned solutions enabled CycIF imaging of ten different components of focal adhesions in fixed REF52 cells. Eight of these components are proteins playing major roles in the structural, mechanical, integrin signaling and actin modulation aspects of focal adhesions—including actin, *α*-actinin, FAK, Hic-5, paxillin, VASP, vinculin and zyxin [1]. These proteins are also located at different positions along the vertical axis of focal adhesions, spanning together from the integrin layer to the actin layer [26]. Therefore, the selected proteins provide collectively an informative and broad sampling of the molecular content of focal adhesions in respect to their assembly and functions. The additional two components are the regulatory phosphorylation sites Y397 of FAK [31] and Y118 of paxillin [32] (Fig 1a). Before the cell fixation and CycIF application, we imaged in a live cell mode the dynamics of all the analyzed focal adhesions using YFP-paxillin as a marker (Fig 1a). Thus, the molecular content of focal adhesions can be correlated with their age and past dynamics. By implementing these CycIF and live cell imaging in a high-throughput manner, thousands of individual focal adhesions were imaged (S1–S3 Figs), obtaining an unprecedented multiplexed quantification of the molecular content of these structures.

### Correlations between components density in focal adhesions

We first evaluated how uniform is the components stoichiometry among focal adhesions. A uniform stoichiometry should be reflected by a strong pair-wise linear correlation between the densities of the components. Two-dimensional scatter plots of protein densities show that the pair-wise relations between the densities of proteins in focal adhesions can be approximated to be either linearly correlated or uncorrelated (S3 Fig). Therefore the strengths and signs of the statistical relations between protein densities can be quantified by Pearson correlation coefficients. Accordingly, the densities of the analyzed components were found to be positively correlated with each other with different strengths (Fig 1b and S3 Fig). This indicates that the instrumental noise is smaller than the biological variations in protein densities, as correlations would be masked otherwise. Based on these results, for some of the analyzed components the stoichiometry in focal adhesions is apparently unconfined, while for the other component pairs the stoichiometry is partially constrained.

The network of pair-wise correlations between the ten components reveal differential degrees of stoichiometry uniformity between the components (Fig 1b). The correlations between the densities of FAK and FAK-pY397, as well as between paxillin and paxillin-pY118, in focal adhesions are moderate, indicating that the relative extent of FAK and paxillin phosphorylation is heterogeneous among focal adhesions. The extent of stoichiometry conservation between each two proteins across focal adhesion does not correlate with the extent of their physical association in the cytosol [33] (Fig 1b). This indicates that the compositional stoichiometry of focal adhesions is actively and locally shaped by them, rather than being a passive reflection of the building blocks design in the cytosolic pool. Strikingly, the strongest correlation is between the densities of zyxin and paxillin (Fig 1b), although these proteins do not interact directly [1, 32] nor physically associate in the cytosol [33] and are located in distal vertical layers across focal adhesions [26]. Zyxin and paxillin appear to form hubs of order within focal adhesions, since proteins that are vertically adjacent to them—FAK, vinculin and VASP [26]—exhibit relatively more conserved stoichiometric ratios among them but not with the distally located *α*-actinin and actin (Fig 1b).

### Changes in noise levels in assembling focal adhesions

**Fig 2:**
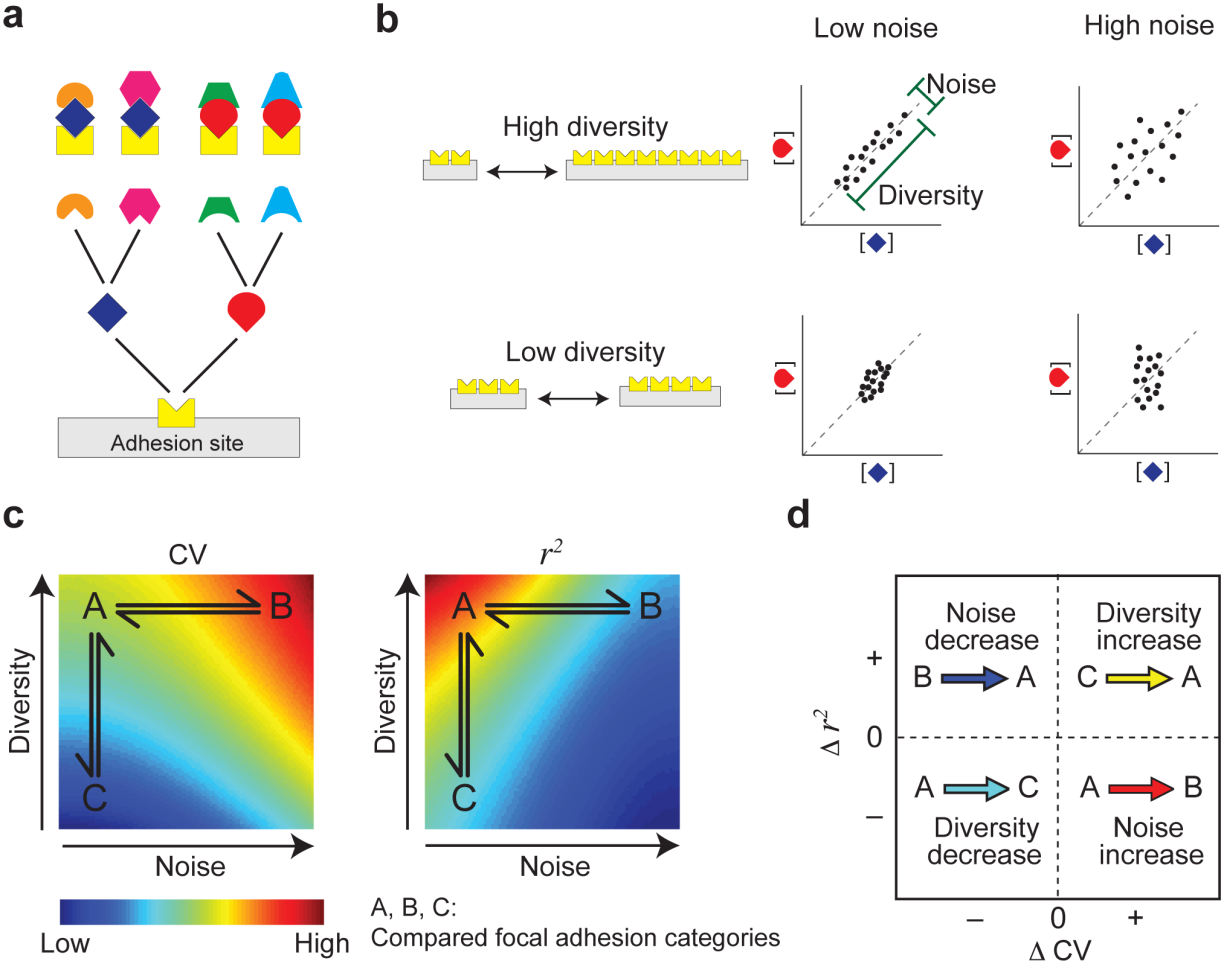
Inferring changes in noise levels in the molecular content of focal adhesions. (a) An assembly process with competing binding interactions. (b) Higher diversity in the local levels of a recruiting protein leads to a stronger correlation between the recruited proteins, while higher noise causes the opposite. (c) Simulated *CV* and *r*^2^ of the densities of the dark-blue and red components as a function of binding noise and diversity in the density of the yellow component among focal adhesions. (d) Inferring changes in noise levels based on Δ*CV* and Δ*r*^2^. Changes between focal adhesion categories exemplified in (c) are indicated. The inference approach was validated by systematic screen of diversity and noise levels for competitive and non-competitive assembly processes.

**Fig 3:**
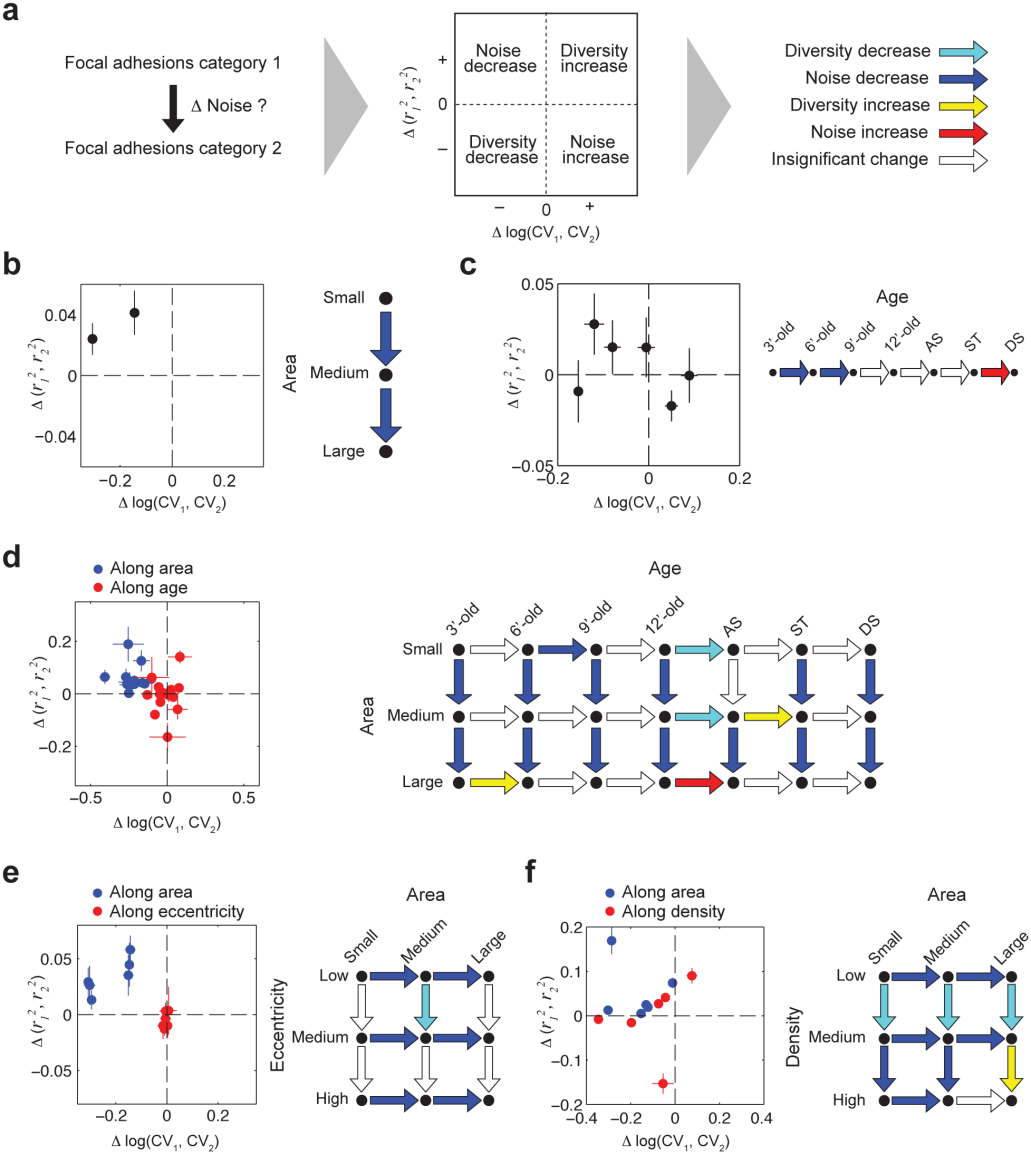
Noise decrease in focal adhesions is coupled to their size and internal density independently. (a) The workflow of inferring changes in noise levels between two categories of focal adhesions (e.g. small versus big focal adhesions). (b) Changes in noise levels as a function of focal adhesions area. (c) Changes in noise levels as a function of focal adhesions age. (d) Focal adhesions were sub-categorized according to both their area and age. Changes in noise levels were inferred within each age category as a function of area and vice versa. (e) Same as (d), using eccentricity instead of age. (f) Same as (d), using density instead of age. Error bars indicate standard error of the mean between datasets (see S1 Fig and S2 Table).

The heterogeneity in the stoichiometry of proteins in focal adhesions can be generated by several factors, including noise, diversity in local cues and distinct responses of the different components to the same cue. Based on the positive linear correlations between the analyzed components in focal adhesions (Fig 1b), we approximated the effective diversity within the analyzed cells as a single parameter that has a common linear effect on the densities of all components (S1 Text)). Noise arises from stochastic alternative recruitment of different proteins to a given recruiting protein while diversity is caused by different levels of cues and maturation phases of focal adhesions (Fig 2a). Both diversity and noise increase the variance of the density of a protein among focal adhesions. However, while diversity-driven variance in the density of a recruiting protein promotes correlations between the recruited proteins, noise-driven variance decreases these correlations (Fig 2a and 2b). As diversity and noise have the same qualitative effect on the variance of protein densities but opposite effects on the correlations between them, changes in noise levels can be untangled. Namely, if between two compared categories of focal adhesions (e.g. small versus big focal adhesions) the coefficient of variation (*CV*) is decreasing and the correlation strength (squared Pearson correlation coefficient, *r*^2^) is increasing, it can be concluded that the noise level is reduced, and vice versa (Figs 2c, 2d, 3a, S4–S7 Figs and S1 Text).

The assembly of focal adhesions is a multistep process, in a sense that recruited proteins often act as recruiters for additional proteins. In such a multistep assembly process, in which each recruiting protein can alternatively recruit different proteins, stochastic noise is expected to accumulate along sequential assembly steps of a structure (S6 Fig). Therefore, without suppressing the effects of stochastic alternative bindings, noise accumulation could drift the assembly of focal adhesions away from its correct paths, thus generating aberrant structures. To assess this, we examined how noise levels change during focal adhesions assembly. First we examined changes in noise levels between focal adhesions with different sizes, since the area of focal adhesions increases during their assembly. The *CV* of the components density persistently decreases as a function of focal adhesions area while the correlations between them increase (Fig 3b and S8 Fig). These changes were observed also if focal adhesions were sampled with equal number of pixels, as a control (S9 Fig). These results indicate that noise in the molecular content of bigger focal adhesions is lower than that of smaller ones (Fig 3b).

We next asked how the noise levels in the molecular content of focal adhesions correlate with their age. Using the live cell imaging data of the focal adhesions prior to the CycIF, focal adhesions were categorized based on their age. Comparison between sequential age categories indicated that during the first 9 minutes of focal adhesions assembly the components density *CV* decreases while their correlations increase, indicating noise reduction (Fig 3c and S10 Fig). In contrast, disassembling focal adhesions exhibit an increase in noise level (Fig 3c). Since the age and area of assembling focal adhesions are positively correlated (S10 Fig), we further examined if each of these parameters is coupled to noise reduction directly or indirectly via the other parameter. For this we sub-categorized the focal adhesions according to both area and age, inferring changes in noise levels along one parameter, keeping the other one fixed (Fig 3d and S11 Fig). The results show that in focal adhesions of the same age category noise is still reduced as a function of their area. In contrast, in focal adhesions of the same area category the noise is not changing as a function of their age (Fig 3d and S11 Fig), indicating that noise reduction is coupled with the area of focal adhesions but not with their age.

A simple model for the observed coupling between focal adhesion area and noise reduction could be based on the reduction in the circumference-to-area ratio of a focal adhesion as it gets bigger. Considering the internal dynamic nature of focal adhesions [34], a lower circumference-to-area ratio would increase the relative frequency of internal interactions between proteins in respect to the frequency of their interactions with soluble cytosolic components. To assess this hypothesis, we examined changes in noise level as a function of the eccentricity of focal adhesions, since a higher eccentricity implies a higher circumference-to-area ratio. However, we found that for focal adhesions of the same area category noise does not change as a function of their eccentricity Fig 3e and S11 Fig), indicating that circumference-to-area ratio is not an important factor for noise reduction. This further raises the possibility that focal adhesions growth is in fact unnecessary for noise suppression. Along this line, the observed correlations between focal adhesions area and noise reduction could actually reflects a dependency of the growth of a focal adhesion on a reduction of noise in its molecular content.

We next tested whether the coupling between focal adhesions area and noise reduction is mediated via the increase in the internal density of focal adhesions as they grow (S12–S14 Figs). For this focal adhesions were sub-categorized based on both their area and internal density, and the *CV* and correlation strengths of protein densities were compared along each of these parameters. Noise was found to decrease as a function of focal adhesions area regardless of changes in their internal density (Fig 3f and S11 Fig). In contrast, in most cases noise was not decreasing as a function of the density per se in focal adhesions of the same size category. Interesting exceptions include the focal adhesions with small or medium area, showing a reduction in noise level between high and medium density categories (Fig 3f, dark-blue vertical arrows). Therefore, although internal density is not ubiquitously coupled with noise suppression in all focal adhesions, it can be coupled with it in subgroups of focal adhesions. Since in these subgroups noise is suppressed as a function of density in focal adhesions of the same area category, we conclude that area and internal density of focal adhesions are coupled with noise reduction by two independent mechanisms.

### High-order relations between components density

The assembly of a conserved multi-molecular structure can rely solely on the selectivity of the interactions between its components. In contrast, for modular assembly of diverse structures with the same components a higher-level of quality control is needed to ensure that the assembly is progressing along the correct path. Considering that focal adhesions are assembled by self-organization of their components without an external supervision of the process, an intriguing question is how quality control is achieved. We hypothesize that a general mechanism for such a quality control would be making the level of each component in a focal adhesion dependent on cues from the levels of multiple other components in that focal adhesion. This can provide robustness to noise, by averaging out stochastic fluctuations in the levels of the cueing components. Moreover, it can guide the system to assemble in a specific path by constraining the alternative interactions of a given protein, for example by generating steric hindrances. Such mechanisms would generate high-order statistical relations between the components, for which the level of a given protein could be statistically modeled better by the consideration of multiple other proteins.

**Fig 4:**
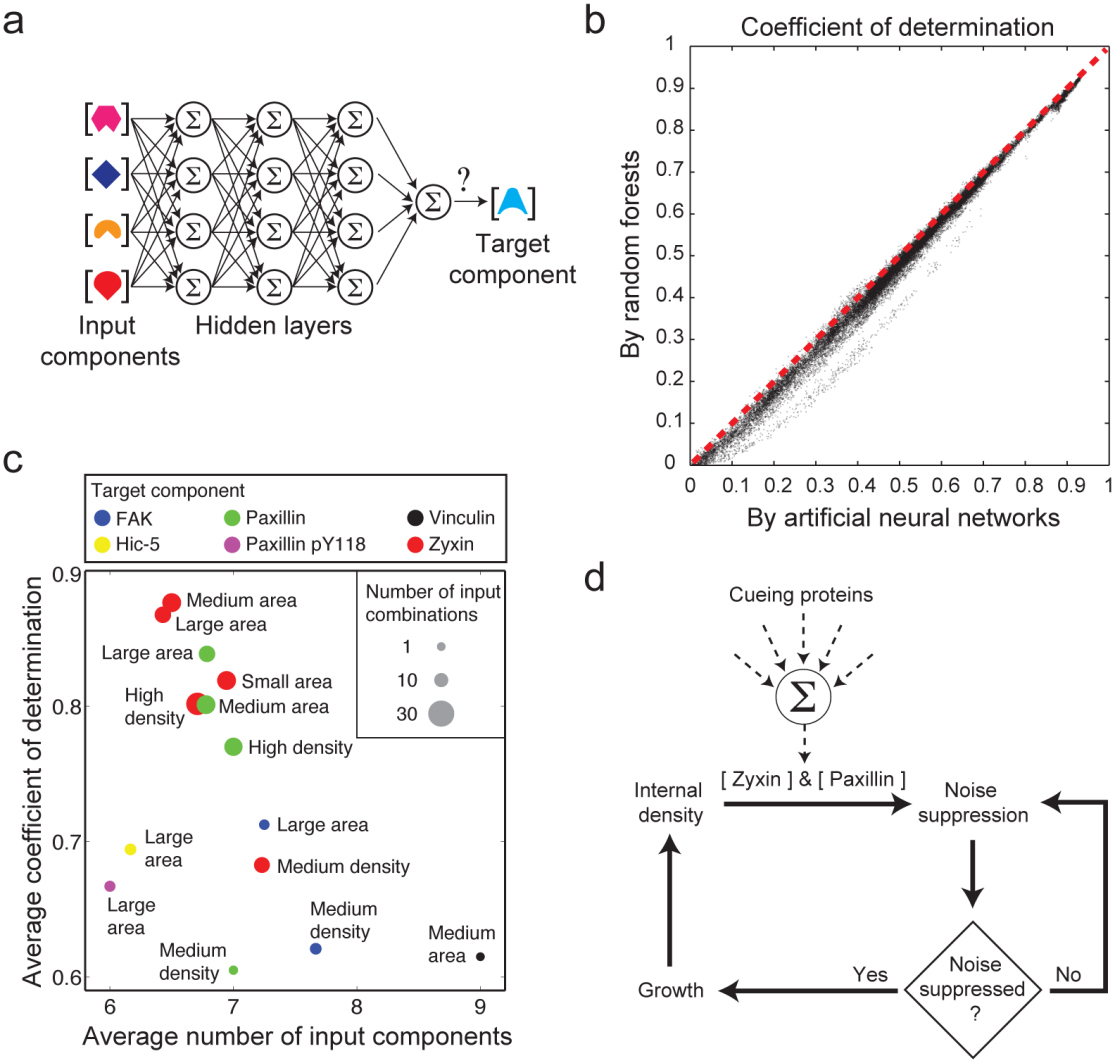
Density-dependent high-order statistical relations between components in focal adhesions. (a) The artificial neural network architecture, exemplified for the case of four input proteins. (b) The coefficients of determinations between the predicted and observed densities of the target component as obtained by artificial neural networks versus Random Forests, shown for all possible combinations of input and target proteins. (c) A scatter plot showing the extent of high-order relations identified for the indicated target components. The horizontal axis indicates the average number of input components in the identified high-order relations. The vertical axis indicates the average coefficient of determination between the predicted levels of the target protein, based on the artificial neural network analysis, and its actual levels in the focal adhesions. High-order relations with average coefficient of determination lower than 0.6 are omitted from the plot. The diameter of the circles indicates the number of identified high-order relations (see Materials and Methods). (d) A positive feedback model for the emergence of noise suppression in focal adhesions.

To reveal high-order statistical relations between the densities of components in focal adhesions, we examined how well artificial neural networks can predict the densities of a target component based on the densities of the other components in these structures (Fig 4a). As a complementary approach, we applied Random Forests analysis [30] to test if hierarchical partitioning of the data according to the densities of a subset of components can provide a good prediction of the densities of a target component in focal adhesions. The coefficients of determination between the observed and predicated densities obtained from the two methods are strongly correlated, with marginally better predictions by artificial neural networks (Fig 4b). A systematic screen of all combinations of input and target components revealed paxillin and zyxin densities to be significantly better predicted upon integration of multiple input components (Fig 4c). Of note, this is not a side effect of the strong pairwise correlation between paxillin and zyxin (Fig 1b) but rather indicates a statistically significant incremental contribution of each of the input components to the prediction of paxillin or zyxin densities (see Materials and Methods). Interestingly, while these high-order relations of zyxin and paxillin are independent of the area of focal adhesions they get more prominent in focal adhesions of higher internal density (Fig 4c). Therefore, high-order relations are plausibly underlying the observed density-coupled suppression of noise in small and medium size focal adhesions (Fig 3f, dark-blue vertical arrows). These results support the hypothesis that the increasing internal density within assembling focal adhesions promotes high-order integration of interactions that tunes the levels of paxillin and zyxin and thus suppresses compositional noise (Fig 4d).

## Discussion

The consideration of noise in the molecular content of adhesion sites assumes a designed optimal level for each protein in each focal adhesion under a given condition. This assumption implies that binding probabilities and protein expression levels are designed to lead, on average, to the optimal level of each protein in each focal adhesion while stochastic noise causes deviations from these optimal levels. Therefore, in the absence of diversity, the effective noise level of a protein in focal adhesions can be defined as the ratio between the amplitude of its stochastic fluctuations over its mean level. According to this definition, noise might be reduced merely due to the increase in the mean copy number of proteins in focal adhesions as they get more dense. However our results show that beside two cases noise is not reduced as a function of the internal density of focal adhesions (Fig 3f). Noise decreases as a function of density only in focal adhesions with small or medium area and only when their internal density increases from medium to high levels (Fig 3f, dark-blue vertical arrows). Although other processes may counteract an existing effect of increase in the protein copy number, these results indicate that such increases do not dominate the inference of changes in noise levels. In addition, image-processing based controls indicate that inferred reductions in noise levels in bigger focal adhesions are not due higher number of sampled pixels, hence also not due increase in the mean copy number of the proteins per focal adhesion (S9 Fig). To further assess this, simulations of assembly processes with stochastic fluctuations that are uncoupled to the mean show that changes in noise levels are correctly inferred (S1 Text). Therefore the observed noise reduction cannot be explained solely by the increase in proteins copy number in assembling focal adhesions, hence pointing toward external processes affecting the noise levels.

Our results suggest that active regulatory processes within focal adhesions dictate their internal molecular organization and reduce its noise. First indication for this is the relatively low correlation between the physical associations of the components in the cytosol and the correlation strengths between their densities in focal adhesions (Fig 1b). Given the combinatorial diversity of the cytosolic building blocks for focal adhesions, many proteins may be recruited to focal adhesions being embedded in different complexes [33]. In such a situation, controlling the molecular content of focal adhesions requires selective recruitment of protein complexes or integration of information within the structure. Most strikingly was the strong correlation between the densities of paxillin and zyxin in focal adhesions (Fig 1b). From the functional perspective, a tight coupling between paxillin and zyxin densities is sensible, as paxillin is a major signaling hub [32, 35] while zyxin modulates actin and is highly responsive to mechanical forces [7, 8, 36, 37]. However, from the mechanistic perspective it is unclear how this coupling is being achieved. Considering the facts that these proteins are not associated with each other in the cytosol [33] and are located at distal vertical layers within the focal adhesion [26], the coupling between them is plausibly mediated via the other proteins within the structure. This hypothesis is supported by our finding that both zyxin and paxillin emerge as prominent target proteins in the artificial neural network screen, suggesting that their densities are particularly tuned by the other proteins in the focal adhesions.

This work suggests that assembling focal adhesions suppress noise in their molecular composition by two mechanisms which are coupled with their area and internal density. These couplings can be mediated by (i) causal effects of focal adhesions area and density on noise suppression, (ii) causal effects of noise suppression on focal adhesions area and internal density, (iii) another factor which affects together the density, area and noise. We excluded the possibility that area increase affects noise reduction via reducing the circumference-to-area ratio. This suggests that the area-noise coupling is mediated by a different direction of causality. It is plausible that noise suppression promotes a stable increase in the area of focal adhesions by configuring correctly cross-linking interactions and minimizing steric hindrances (Fig 4d). In regards to the density-noise coupling we found that high-order interactions, which can facilitate noise suppression, get more prominent in focal adhesions with higher internal density (Fig 4c). We suggest that coupling between internal density and noise suppression is generated by a positive causal effect of the former one on the latter one (Fig 4d). These two causal relations form a feedback loop between the internal density, noise suppression and growth of a focal adhesion which gives rise to a quality control of their assembly and maintenance (Fig 4d). According to this model, as a focal adhesion assembles the increase in its internal density (S12–S14 Figs) facilitates noise suppression, which in turn enables its further stable growth in area and thereby a further increase in internal density.

## Supporting Information

S1 Text Simulation details and assessment of the inference method. (PDF)

S1 Fig The number of focal adhesions in the different datasets and focal adhesion categories. (a) The number of cells and focal adhesions in each dataset. (b) The number of focal adhesions in each area and age category. (c) Tables showing the number of focal adhesions in each sub-category, as indicated. The numbers within each rectangle correspond from top to bottom to dataset R1O1, R1O2, R2O1, R2O2, R3O1 and R3O2.
(PDF)

S2 Fig Identification of the analyzed adhesion sites as focal adhesions. (a) The percentage of adhesion sites that do not contain detectable levels a given component (mean ± standard error of the mean, *n* = 6 datasets). Note that almost all adhesion sites contain all of the components, with negligible exceptions plausibly due to thresholding effects. The presence of zyxin indicates that these sites are focal adhesions rather than focal-complexes. (b) The distribution of the eccentricities of the adhesion sites indicates that they are oval, further supporting that they are focal adhesions and not focal complexes. (c) The distribution of the eccentricities of the adhesion sites with undetectable zyxin levels, showing that also most of those sites are oval.
(PDF)

S3 Fig Scatterplots and histograms of the components normalized densities in the focal adhesions. For clarity, each scatterplot shows a random sample of 0.1% from all focal adhesions of the indicated dataset.
(TIF)

S4 Fig Testing noise inference with simulated competitive assembly process. (a) The model and its letter notation. The level of component *A* is varying among sites due to diversity in local cues. (b) The total level, P^*T*^, of a given protein, *P*, in an adhesion site is the sum of its levels in all its assemblies there. (c) The model was simulated with different noise and diversity levels, as described. For each possible pair of simulated noise and diversity levels, the signs of changes in these levels were inferred based on the Δ*r*^2^ between the total levels of the indicated components and Δ*CV* of the first one. Red bars show the fraction of observations in each category. Blue stripes indicate the expected possible changes in noise and diversity based on the inference rules (Fig 2d).
(PDF)

S5 Fig Testing noise inference with simulated non-competitive assembly process. (a) The model and its letter notation. The level of component *A* is varying among sites due to diversity in local cues. (b) The total level, *P*^*T*^, of a given protein, *P*, in an adhesion site is the sum of its levels in all its assemblies there. (c) The model was simulated with different noise and diversity levels, diversity mean/range ratios and equal or unequal binding sites for proteins *B* and *C*, as described. For each possible pair of simulated noise and diversity levels, the signs of changes in these levels were inferred based on the Δ*r*^2^ between *A*^*T*^ and *B*^*T*^ and Δ*CV* of *A*^*T*^. Red bars show the fraction of observations in each category. Blue stripes indicate the expected possible changes in noise and diversity based on the inference rules (Fig 2d). (d) As (c), using different diversity distributions as described.
(PDF)

S6 Fig Noise increase along the steps of a sequential assembly process. (a) The simulated model, consisting of two layers of non-competitive interactions. The level of component *A* is varying from site to site due to diversity in local cues. (b) The noise inference scheme. (c) Changes in Δ*log*(*CV*) and Δ(*r*^2^) between proteins recruited in the first assembly step (category 1) and those recruited in the second step (category 2). (d) Inferred changes in noise levels between the sequential assembly steps.
(PDF)

S7 Fig Comparison between the change in *CV* and the change the Fano factor (variance/mean) between the sub-categories of focal adhesions that were compared in this study. Note that beside few exceptions, the changes in these two measures have the same sign. Error bars indicate standard error of the mean between the datasets (see S1 Fig and S2 Table).
(PDF)

S8 Fig Changes in the components density, *CV* and *r*^2^ as a function of focal adhesions area. (a) The mean densities (*n* = 6 datasets) of the labeled components and their *CV* as a function of focal adhesions area. (b) Scatter plots comparing the *r*^2^ between the components in focal adhesions between the area categories. Error bars indicate standard error of the mean (*n* = 6 datasets). (c) Δ*log*(*CV*) and Δ(*r*^2^) as a function of focal adhesions area.
(PDF)

S9 Fig The reduction in noise level in bigger focal adhesions is not due to the higher number of pixels per focal adhesion. (a) Inferring changes in noise levels as a function of focal adhesions area based on Δ*log*(*CV*) and Δ(*r*^2^). However, here, the densities of proteins in each focal adhesion in the medium and large size categories were calculated using only a randomly sampled fraction of the pixels, such that the mean number of pixels sampled per focal adhesion is equal among all area categories. (b) The same as (a), but with sampling an equal number of pixels (10 pixels) from each focal adhesion in all area categories. Note that in both (a) and (b) the number of pixels used for calculating the *CV* and *r*^2^ is equal for all compared area categories, yet without affecting the detection of the reduction in the noise level. Error bars indicate standard error of the mean (*n* = 6 datasets).
(PDF)

S10 Fig Changes in the components density, *CV* and *r*^2^ as a function of focal adhesions age. (a) The total intensity of YFP-paxillin during the last 12 minutes before fixation in individual, randomly sampled, focal adhesions of the different age categories (b) The areas of focal adhesions in each age category. Error bars denote standard deviation (*n* = 6 datasets). (c) The mean densities and *CV* (*n* = 6 datasets) of the labeled components as a function of focal adhesions age. (d) Scatter plots comparing *r*^2^ between the component densities in focal adhesions of sequential age categories. Error bars indicate standard error of the mean (*n* = 6 datasets). (e) Δ*log*(*CV*) and Δ(*r*^2^) as a function of focal adhesions age.
(PDF)

S11 Fig Changes in *CV* and *r*^2^ among sub-categorized focal adhesions. (a) Focal adhesions were sub-categorized according to both their area and age. Δ*log*(*CV*) and Δ(*r*^2^) were calculated between focal adhesions of the same age category as a function of area, as well as between focal adhesions of the same area category as a function of age. (b) As (a), using density instead of age. (c) As (a), using eccentricity instead of age.
(PDF)

S12 Fig The average Pearson correlation coefficient (*r*) between the area of focal adhesions and the corresponding densities of the components in these focal adhesions. Error bars indicate standard error of the mean (*n* = 6 datasets).
(PDF)

S13 Fig (a) The mean, variance and *CV* of the density of the various components in focal adhesions of the different area categories and in the different datasets. S, M and L denote the small, medium and large area categories, respectively. Error bars indicate standard error of the mean (see S1 Fig for the number of analyzed focal adhesions for each dataset and area category). (b) The mean Pearson correlation, *r*, (*n* = 6 datasets) between the densities of the components in focal adhesions of the different area categories.
(PDF)

S14 Fig (a) The mean, variance and *CV* of the density of the various components in focal adhesions of the different age categories and in the different datasets. Error bars indicate standard error of the mean (see S1 Fig for the number of analyzed focal adhesions for each dataset and age category). (b) The mean Pearson correlation, *r*, (*n* = 6 datasets) between the densities of the components in focal adhesions of the different age categories.
(PDF)

S1 Table The two labeling orders of the components in the CycIF cycles. (PDF)

S2 Table Supporting data for Figs 1 and 3 and S7 Fig. (XLSX)

## References

[1] Zaidel-Bar R, Itzkovitz S, Ma’ayan A, Iyengar R, Geiger B. Functional atlas of the integrin adhesome. Nat Cell Biol. 2007 8;9(8):858–67. 10.1038/ncb0807-858 17671451 PMC2735470

[2] Zaidel-Bar R, Geiger B. The switchable integrin adhesome. J Cell Sci. 2010 5;123(Pt 9):1385–8. 10.1242/jcs.066183 20410370 PMC2858016

[3] Zamir E, Katz BZ, Aota S, Yamada KM, Geiger B, Kam Z. Molecular diversity of cell-matrix adhesions. J Cell Sci. 1999 6;112:1655–69. 10318759 10.1242/jcs.112.11.1655

[4] Zamir E, Katz M, Posen Y, Erez N, Yamada KM, Katz BZ, et al Dynamics and segregation of cell-matrix adhesions in cultured fibroblasts. Nat Cell Biol. 2000 4;2(4):191–6. 10.1038/35008607 10783236

[5] Zamir E, Geiger B. Molecular complexity and dynamics of cell-matrix adhesions. J Cell Sci. 2001 10;114(Pt 20):3583–90. 11707510 10.1242/jcs.114.20.3583

[6] Katz BZ, Zamir E, Bershadsky A, Kam Z, Yamada KM, Geiger B. Physical state of the extracellular matrix regulates the structure and molecular composition of cell-matrix adhesions. Mol Biol Cell. 2000 3;11(3):1047–60. 10.1091/mbc.11.3.1047 10712519 PMC14830

[7] Lavelin I, Wolfenson H, Patla I, Henis YI, Medalia O, Volberg T, et al Differential effect of actomyosin relaxation on the dynamic properties of focal adhesion proteins. PLoS One. 2013;8(9):e73549 10.1371/journal.pone.0073549 24039980 PMC3767655

[8] Wolfenson H, Bershadsky A, Henis YI, Geiger B. Actomyosin-generated tension controls the molecular kinetics of focal adhesions. J Cell Sci. 2011 5;124(Pt 9):1425–32. 10.1242/jcs.077388 21486952 PMC3078811

[9] Zamir E, Geiger B, Kam Z. Quantitative multicolor compositional imaging resolves molecular domains in cell-matrix adhesions. PLoS One. 2008;3(4):e1901 10.1371/journal.pone.0001901 18382676 PMC2270910

[10] Kam Z, Zamir E, Geiger B. Probing molecular processes in live cells by quantitative multidimensional microscopy. Trends Cell Biol. 2001 8;11(8):329–34. 10.1016/S0962-8924(01)02067-0 11489638

[11] Grecco HE, Imtiaz S, Zamir E. Multiplexed imaging of intracellular protein networks. Cytometry A. 2016 5 10.1002/cyto.a.22876 27183498

[12] Robertson J, Jacquemet G, Byron A, Jones MC, Warwood S, Selley JN, et al Defining the phospho-adhesome through the phosphoproteomic analysis of integrin signalling. Nat Commun. 2015;6:6265 10.1038/ncomms7265 25677187 PMC4338609

[13] Geiger T, Zaidel-Bar R. Opening the floodgates: proteomics and the integrin adhesome. Curr Opin Cell Biol. 2012 10;24(5):562–8. 10.1016/j.ceb.2012.05.004 22728062

[14] Römpp A, Spengler B. Mass spectrometry imaging with high resolution in mass and space. Histochem Cell Biol. 2013 6;139(6):759–83. 10.1007/s00418-013-1097-6 23652571 PMC3656243

[15] Friedenberger M, Bode M, Krusche A, Schubert W. Fluorescence detection of protein clusters in individual cells and tissue sections by using toponome imaging system: sample preparation and measuring procedures. Nat Protoc. 2007;2(9):2285–94. 10.1038/nprot.2007.320 17853885

[16] Schubert W. Topological proteomics, toponomics, MELK-technology. Adv Biochem Eng Biotechnol. 2003;83:189–209. 12934931 10.1007/3-540-36459-5_8

[17] Schubert W, Bonnekoh B, Pommer AJ, Philipsen L, Böckelmann R, Malykh Y, et al Analyzing proteome topology and function by automated multidimensional fluorescence microscopy. Nat Biotechnol. 2006 10;24(10):1270–8. 10.1038/nbt1250 17013374

[18] Schubert W. Exploring molecular networks directly in the cell. Cytometry A. 2006 3;69(3):109–12. 10.1002/cyto.a.20234 16496422

[19] Zrazhevskiy P, True LD, Gao X. Multicolor multicycle molecular profiling with quantum dots for single-cell analysis. Nat Protoc. 2013 10;8(10):1852–69. 10.1038/nprot.2013.112 24008381 PMC4108347

[20] Lin JR, Fallahi-Sichani M, Sorger PK. Highly multiplexed imaging of single cells using a high-throughput cyclic immunofluorescence method. Nat Commun. 2015;6:8390 10.1038/ncomms9390 26399630 PMC4587398

[21] Schubert W. Systematic, spatial imaging of large multimolecular assemblies and the emerging principles of supramolecular order in biological systems. J Mol Recognit. 2014 1;27(1):3–18. 10.1002/jmr.2326 24375580 PMC4283051

[22] Schubert W. Advances in toponomics drug discovery: Imaging cycler microscopy correctly predicts a therapy method of amyotrophic lateral sclerosis. Cytometry A. 2015 8;87(8):696–703. 10.1002/cyto.a.22671 25869332 PMC4676937

[23] Guizar-Sicairos M, Thurman ST, Fienup JR. Efficient subpixel image registration algorithms. Opt Lett. 2008;33:156–158. 10.1364/OL.33.000156 18197224

[24] Zaidel-Bar R, Ballestrem C, Kam Z, Geiger B. Early molecular events in the assembly of matrix adhesions at the leading edge of migrating cells. J Cell Sci. 2003 11;116(Pt 22):4605–13. 10.1242/jcs.00792 14576354

[25] Zaidel-Bar R, Cohen M, Addadi L, Geiger B.Hierarchical assembly of cell-matrix adhesion complexes. Biochem Soc Trans. 2004 6;32(Pt3):416–20. 10.1042/bst0320416 15157150

[26] Kanchanawong P, Shtengel G, Pasapera AM, Ramko EB, Davidson MW, Hess HF, et al Nanoscale architecture of integrin-based cell adhesions. Nature. 2010 11;468(7323):580–4. 10.1038/nature09621 21107430 PMC3046339

[27] Box GEP, Cox DR. An Analysis of Transformations. J R Stat Soc Ser B Stat Methodol. 1964;26(2):211–252.

[28] Tukey JW. Exploratory Data Analysis. Addison-Wesley, Reading, MA; 1977.

[29] Otsu N. A threshold selection method from gray-level histograms. IEEE Trans Sys, Man, Cyber. 1979;9(1):62–66. 10.1109/TSMC.1979.4310076

[30] Breiman L. Random Forests. Machine Learning. 2001;45:5–32. 10.1023/A:1010933404324

[31] Mitra SK, Hanson DA, Schlaepfer DD. Focal adhesion kinase: in command and control of cell motility. Nat Rev Mol Cell Biol. 2005 1;6(1):56–68. 10.1038/nrm1549 15688067

[32] Deakin NO, Turner CE. Paxillin comes of age. J Cell Sci. 2008 8;121(Pt 15):2435–44. 10.1242/jcs.018044 18650496 PMC2522309

[33] Hoffmann JE, Fermin Y, Stricker RL, Ickstadt K, Zamir E. Symmetric exchange of multi-protein building blocks between stationary focal adhesions and the cytosol. Elife. 2014;3:e02257 24894463 10.7554/eLife.02257 PMC4040925

[34] Rossier O, Octeau V, Sibarita JB, Leduc C, Tessier B, Nair D, et al Integrins *β*1 and *β*3 exhibit distinct dynamic nanoscale organizations inside focal adhesions. Nat Cell Biol. 2012 10;14(10):1057–67. 10.1038/ncb2588 23023225

[35] Turner CE. Paxillin interactions. J Cell Sci. 2000 12;113 Pt 23:4139–40. 11069756 10.1242/jcs.113.23.4139

[36] Hirata H, Tatsumi H, Sokabe M. Zyxin emerges as a key player in the mechanotransduction at cell adhesive structures. Commun Integr Biol. 2008;1(2):192–5. 10.4161/cib.1.2.7001 19513257 PMC2686020

[37] Lele TP, Pendse J, Kumar S, Salanga M, Karavitis J, Ingber DE. Mechanical forces alter zyxin unbinding kinetics within focal adhesions of living cells. J Cell Physiol. 2006 4;207(1):187–94. 10.1002/jcp.20550 16288479

