# Supplementary material for: Highly Multiplexed Imaging Uncovers Changes in Compositional Noise within Assembling Focal Adhesions
Source: PLoS One. 2016 Aug 12;11(8):e0160591. doi: 10.1371/journal.pone.0160591 (PMC4982658; doi:10.1371/journal.pone.0160591)
Supplement: S1 Text — (PDF) [file pone.0160591.s001.pdf]

Supplementary Text S1

Simulation of competitive assembly model

Simulation of a multistep assembly process with competing interactions was performed as shown in Supplementary Figure S4. In each step, two distinct proteins (x and y) compete for the same N identical and independent binding sites (B) in M focal adhesions. The resulting fractions of bounded x (BX) and y (BY) and free binding sites were calculated according to mass action in steady state. Biological diversity in the number of binding sites among the focal adhesions was introduced by assigning for each focal adhesion, M_j, a number of binding sites, N_j, sampled from a uniform or normal distribution, where the width of these distributions reflects the level of diversity among the M focal adhesions. Biochemical noise was introduced by defining for each focal adhesion two values ($T_{1,j}, T_{2,j}$) and sampling a random value v_j from a uniform distribution between 0 and 1. The site was considered either as occupied by protein x if $v_j < T_{1,j}$, occupied by protein y if $v_j > T_{2,j}$ or empty if $T_{1,j} < v_j < T_{2,j}$. The values for $T_{1,j}$ and $T_{2,j}$ were sampled from normal distributions centered around 0.3 and 0.6, respectively. The standard deviation of these distributions was scanned from 0.2 (low noise) to 0.5 (high noise). This procedure was then repeated for the two branches of the next layer, using BX or BY as the new numbers of binding sites. For each diversity and noise values, we simulated the occupancy by x or y for one million focal adhesions.

Simulation of non-competitive assembly model

Simulation of an assembly process with multivalent, non-competitive interactions was performed as shown in Supplementary Figure S5. In each step, two distinct proteins (x and y) can bind with probability P_x and P_y to N_x and N_y identical and independent binding sites (B) in M focal adhesions. The resulting fractions of bounded x (BX) and y (BY) were obtained as a realization of binomial distributions with the corresponding binding probabilities and number of binding sites. Biological diversity in the number of binding sites among the focal adhesions was introduced by assigning to each focal adhesion j, a certain size S sampled from a discrete uniform distribution with mean value M and fractional half width W. W reflects the level of diversity among the focal adhesions. The numbers of binding sites for x and y were drawn from a distribution with a mean equal to S. We tested different distributions for the generation of diversity (Supplementary Fig. S5), including using directly S (referred as "uniform"), Poisson distribution (referred as "Poisson(uniform)") and normal distribution with standard deviation equal to one (referred as "normal(uniform)"). Biochemical noise in the binding probabilities was introduced by sampling for each focal adhesion two values ($P_{X,j}, P_{Y,j}$) from a uniform distribution centered around 0.5. The width N of this distribution reflects the level of noise among the focal adhesions. M was scanned from 500 to 1500 and W from 0.05 (low diversity) to 0.95 (high diversity). N was scanned from 0.01 (low noise) to 0.5 (high noise). For each diversity and noise values, the occupancy by x or y was simulated for half a million focal adhesions.

Calculation of observational parameters

As two observational parameters we calculated the r^2 between the amount of any pair of proteins and the CV of the amount of each protein across the focal adhesions. Since the observational data cannot distinguish in which complex the proteins are embedded, we calculated an observed protein amount as the sum over all the complexes in which it is involved (Supplementary Figs. S4b, S5b). All calculations and simulations were performed using Python 2.7.9, NumPy 1.9.0 and SciPy 0.14.

Using variance and correlations to untangle diversity and noise

Focal adhesions in different age or area categories were found to have different internal densities of their components (Supplementary Figs. S13a, S14a), indicating biological diversity in the density of binding sites among focal adhesions. Such diversity enables to detect the correlations between the densities of the components (Fig. 1 and Fig. 2). Since all correlations were found to be positive (Fig. 1b), we assume that the dominating mechanism underlying them is based on common recruiting proteins for multiple proteins, as featured in the assembly models (Fig. 2a, Supplementary Figs. S4, S5). This assumption is also consistent with the large number of mutual exclusive interactions, as well as nonexclusive multivalent interactions, in the integrin adhesome [1, 2, 3, 4]. Thus, proteins that are recruited via the same recruiting protein will be positively correlated, given diversity in the density of the recruiting protein among focal adhesions. Within each category, the variance of the densities can be caused by both intra-category noise and intra-category diversity. However, while intra-category diversity enables and increases the observed correlations (Supplementary Figs. S13b, S14b), intra-category noise decreases it. Therefore, if between two compared sub-categories r^2 is increasing while the CV is not increasing, it can be concluded that the noise level is decreasing, and vice versa (Fig. 2).

Validation of the inference method

We tested the above-mentioned concepts by simulating different variants of competitive and noncompetitive assembly processes (Supplementary Figs. S4, S5) as described above. Noise was modulated by changing the variance of the binding probabilities. Diversity was modulated by changing the variance of the number of binding sites among focal adhesions, first in respect to a constant mean and then scanning the mean value as well. When this mean value was changed we also tested the fractional diversity as change in variance with respect to its mean value. We randomly subsampled 10^5 pairs of simulated points, establishing for each pair to which of the four simulation group (increase or decrease of noise and diversity) they belonged. Additionally we calculated the observational parameters $\Delta log(CV_1, CV_2)$ and $\Delta(r_1^2, r_2^2)$. Since in our experimental data the mean internal density of focal adhesions is changing between the categories, we further considered two measures to quantify the relation between the variance and the mean: CV (standard deviation/mean) and Fano factor (variance/mean). Mathematically, $\Delta log(CV_1, CV_2)$ and $\Delta log(Fano_1, Fano_2)$ may get opposite signs for the same compared data. In such cases, the results of the above-mentioned noise inference procedure would not have converged. However, in our experimental data, the sign of $\Delta log(CV_1, CV_2)$ is as

of $\Delta log(Fano_1, Fano_2)$, beside few exceptions (Supplementary Fig. S7). Therefore, we extracted from the simulation only cases in which $\Delta log(CV_1, CV_2)$ and $\Delta log(Fano_1, Fano_2)$ get the same sign. In all those cases, changes in noise levels were indeed correctly inferred according to the inference procedure (Supplementary Figs. S4, S5).

References

[1] Köster J, Zamir E, Rahmann S. Efficiently mining protein interaction dependencies from large text corpora. Integr Biol (Camb). 2012 Jul;4(7):805–12.

[2] Zaidel-Bar R, Itzkovitz S, Ma'ayan A, Iyengar R, Geiger B. Functional atlas of the integrin adhesome. Nat Cell Biol. 2007 Aug;9(8):858–67.

[3] Zaidel-Bar R, Geiger B. The switchable integrin adhesome. J Cell Sci. 2010 May;123(Pt 9):1385–8.

[4] Zamir E, Geiger B. Molecular complexity and dynamics of cell-matrix adhesions. J Cell Sci. 2001 Oct;114(Pt 20):3583–90.
