# Supplementary material for: Highly Multiplexed Imaging Uncovers Changes in Compositional Noise within Assembling Focal Adhesions
Source: PLoS One. 2016 Aug 12;11(8):e0160591. doi: 10.1371/journal.pone.0160591 (PMC4982658; doi:10.1371/journal.pone.0160591)
Supplement: S1 Fig — (a) The number of cells and focal adhesions in each dataset. (b) The number of focal adhesions in each area and age category. (c) Tables showing the number of focal adhesions in each sub-category, as indicated. The numbers within each rectangle correspond from top to bottom to dataset R1O1, R1O2, R2O1, R2O2, R3O1 and R3O2. (PDF) [file pone.0160591.s002.pdf]

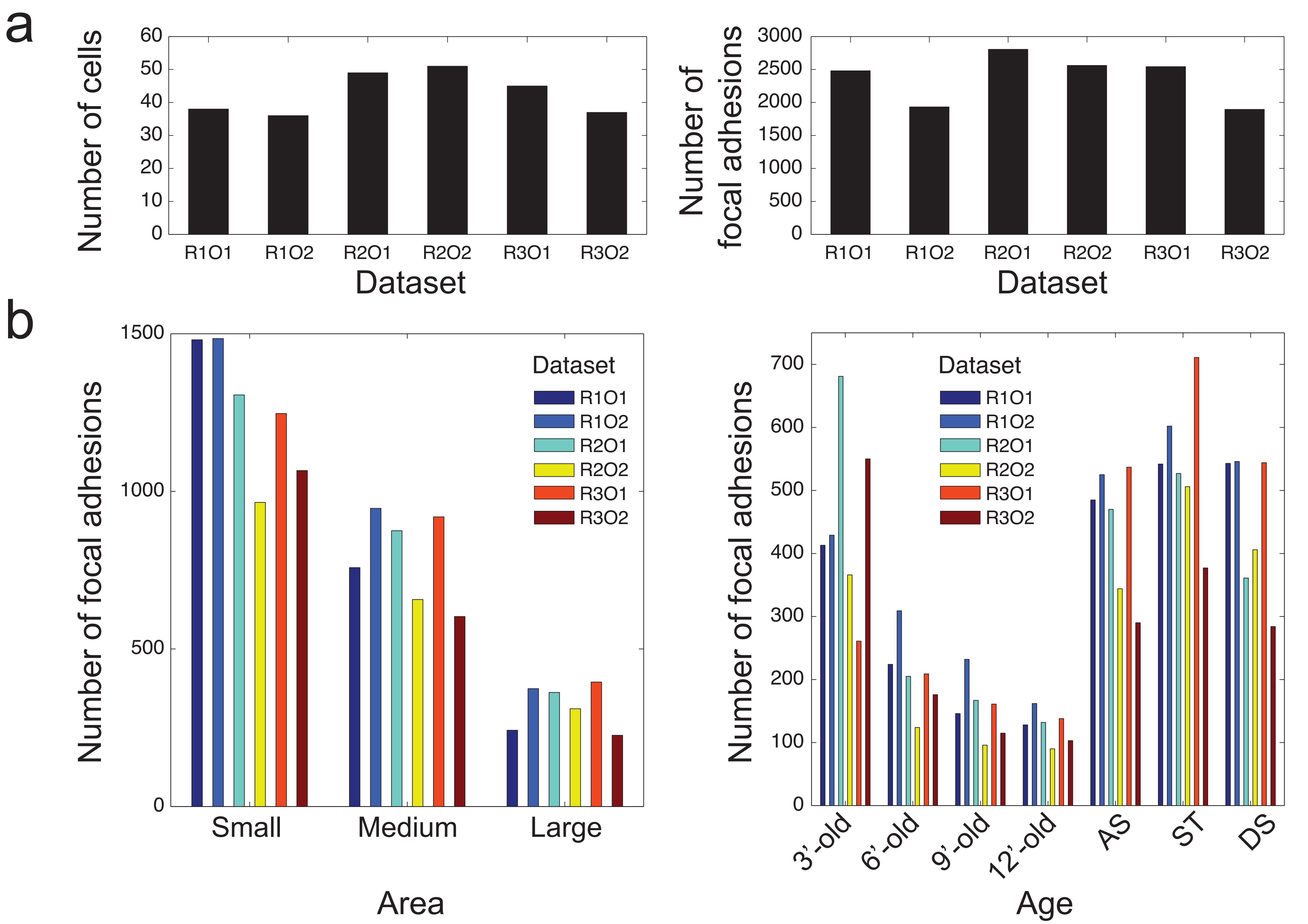

c

Age

Area		3'-old	6'-old	9'-old	12'-old	AS	ST	DS
Small		391	171	113	97	147	256	306
		386	235	138	86	121	235	284
		605	144	105	62	81	147	162
		330	92	57	50	86	177	173
		220	167	114	94	137	262	253
		474	123	78	51	71	130	139
Medium		21	45	29	29	241	204	189
		33	62	84	58	272	243	194
		66	52	54	52	246	261	144
		28	22	33	34	155	221	164
		37	34	41	39	247	308	213
		62	44	33	44	139	171	110
Large		1	8	4	2	97	82	48
		10	12	10	18	132	124	68
		10	9	8	18	143	119	55
		8	10	6	6	103	108	69
		4	8	6	5	153	141	78
		14	9	4	8	80	76	35

Eccentricity

Area	Low	Medium	High
Small	837	356	288
	780	384	321
	706	294	306
	535	240	190
	611	326	310
	573	254	239
Medium	353	178	227
	540	201	205
	463	212	200
	350	169	138
	418	251	250
	298	166	139
Large	117	63	62
	205	83	86
	170	108	84
	174	76	60
	196	103	96
	113	59	54

Internal density

Area	Low	Medium	High
Small	391	171	113
	386	235	138
	605	144	105
	330	92	57
	220	167	114
	474	123	78
Medium	21	45	29
	33	62	84
	66	52	54
	28	22	33
	37	34	41
	62	44	33
Large	1	8	4
	10	12	10
	10	9	8
	8	10	6
	4	8	6
	14	9	4
