# Supplementary figures and images for: Highly Multiplexed Imaging Uncovers Changes in Compositional Noise within Assembling Focal Adhesions
Source: PLoS One. 2016 Aug 12;11(8):e0160591. doi: 10.1371/journal.pone.0160591 (PMC4982658; doi:10.1371/journal.pone.0160591)

**a**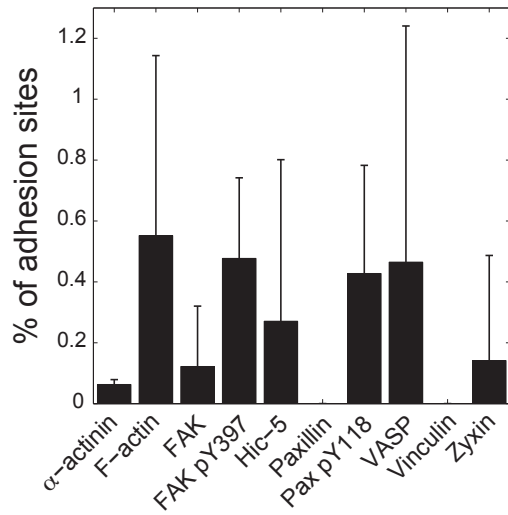**b**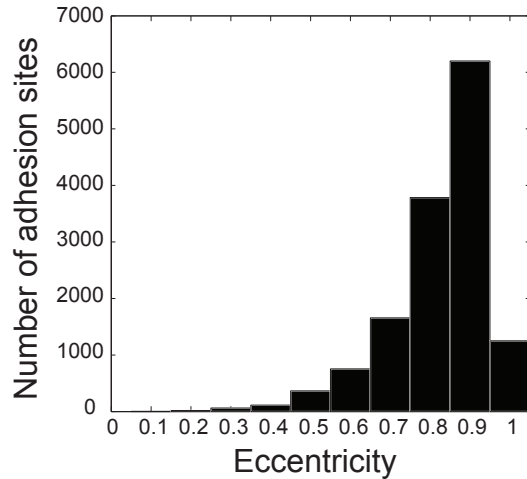**c**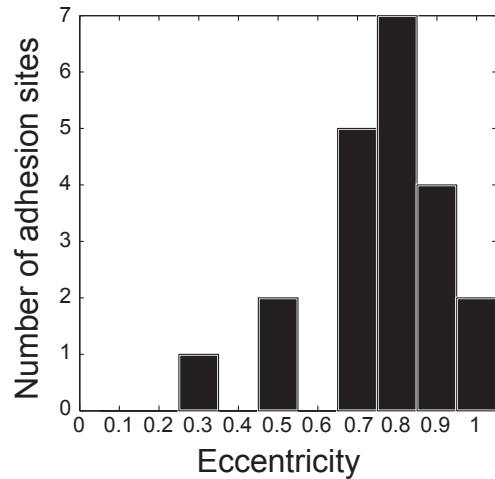

Supplement: S2 Fig — (a) The percentage of adhesion sites that do not contain detectable levels a given component (mean ± standard error of the mean, n = 6 datasets). Note that almost all adhesion sites contain all of the components, with negligible exceptions plausibly due to thresholding effects. The presence of zyxin indicates that these sites are focal adhesions rather than focal-complexes. (b) The distribution of the eccentricities of the adhesion sites indicates that they are oval, further supporting that they are focal adhesions and not focal complexes. (c) The distribution of the eccentricities of the adhesion sites with undetectable zyxin levels, showing that also most of those sites are oval. (PDF) [file pone.0160591.s003.pdf]

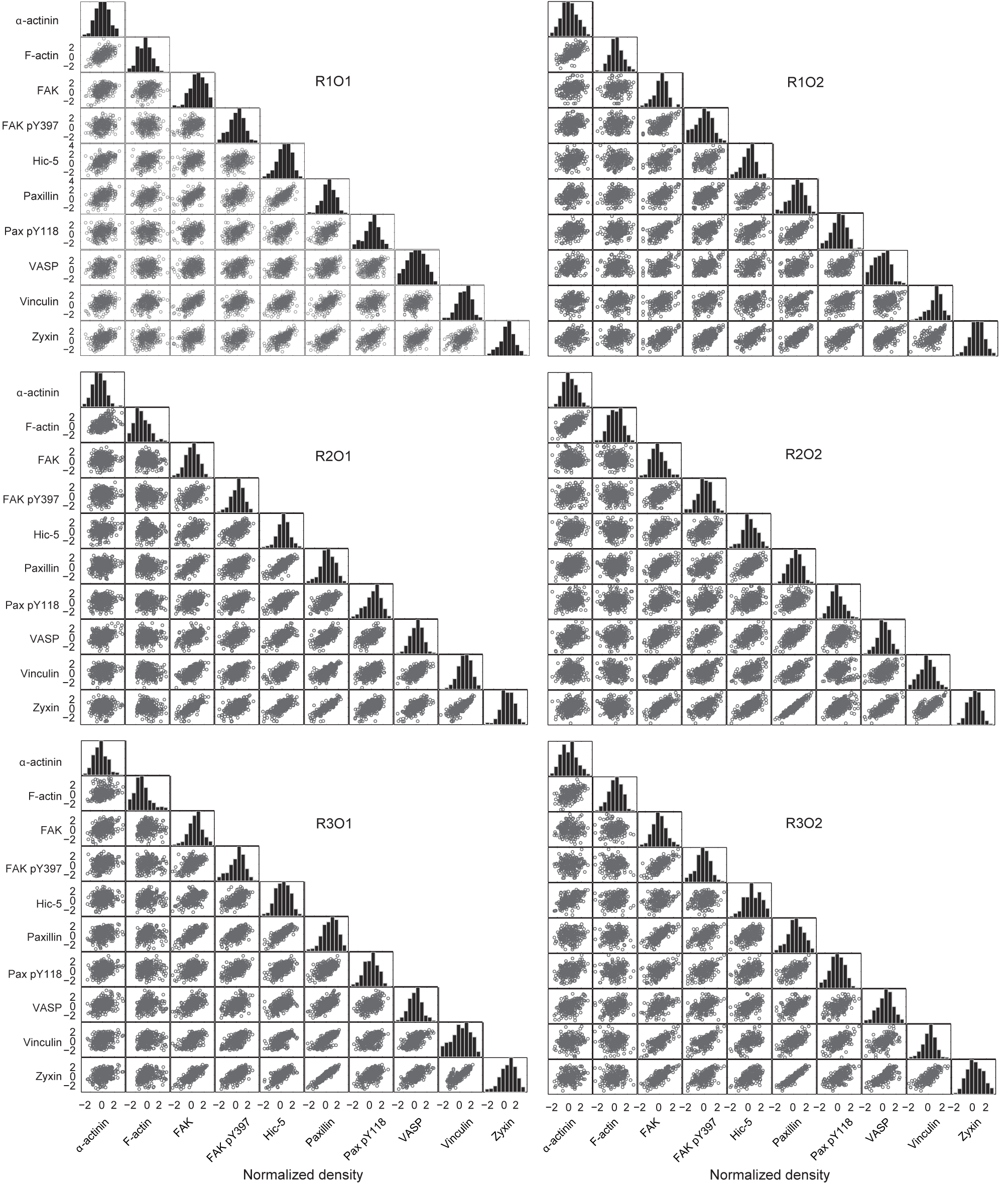

Supplement: S3 Fig — For clarity, each scatterplot shows a random sample of 0.1% from all focal adhesions of the indicated dataset. (TIF) [file pone.0160591.s004.tif]

a

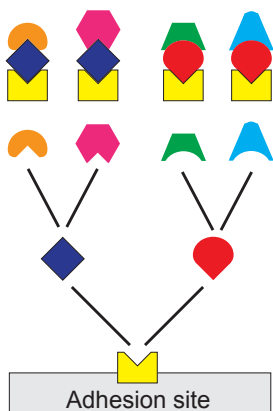

b

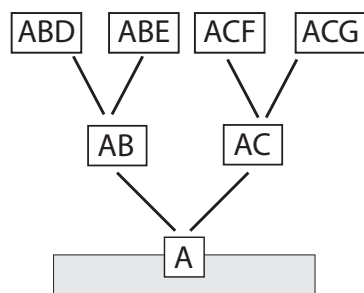

$$A^T = A + AB + AC + ABD + ABE + AC + ACF + ACG$$

$$B^T = AB + ABD + ABE$$

$$C^T = AC + ACF + ACG$$

$$D^T = ABD \quad F^T = ACF$$

$$E^T = ABE \quad G^T = ACG$$

c

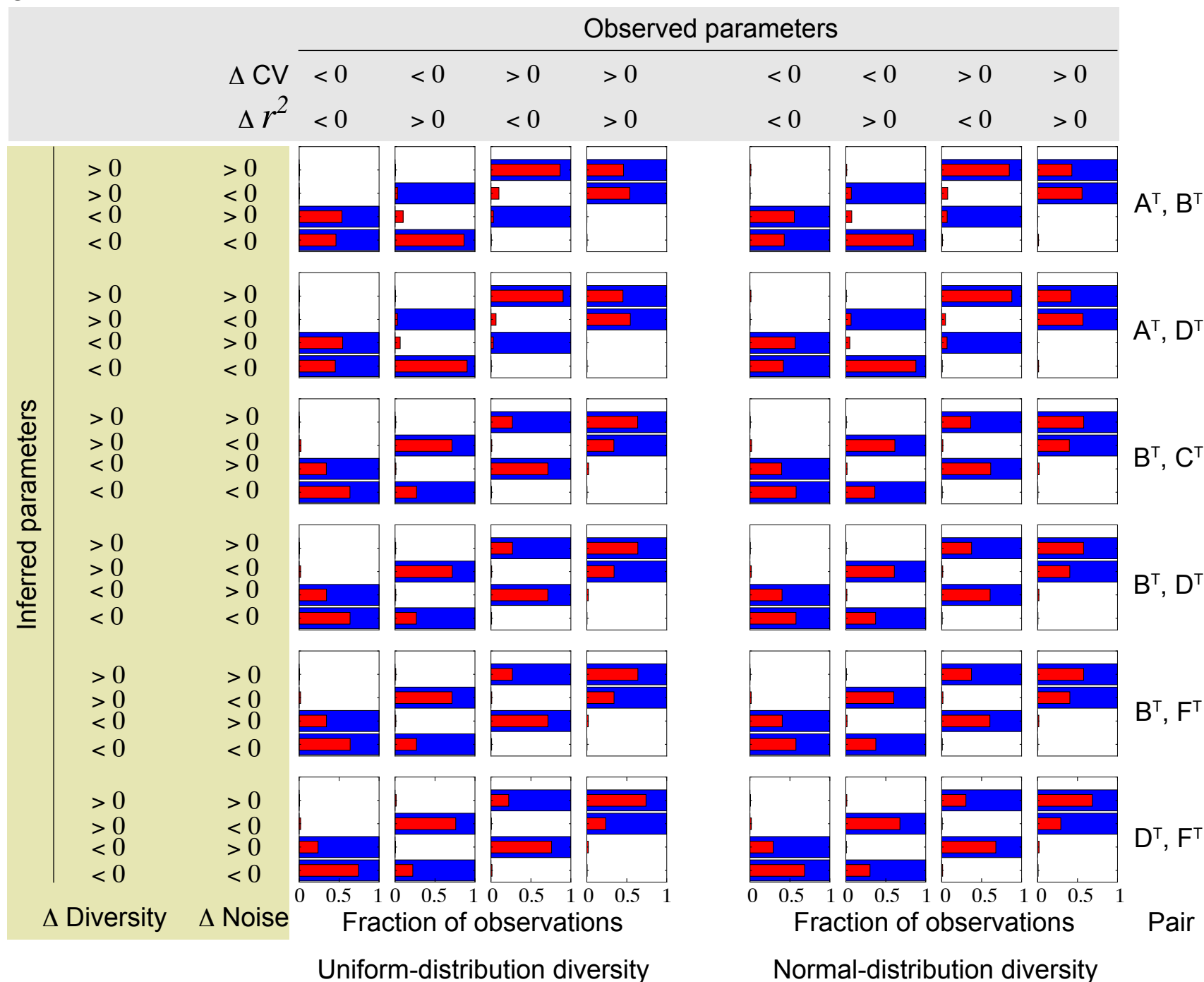

Supplement: S4 Fig — (a) The model and its letter notation. The level of component A is varying among sites due to diversity in local cues. (b) The total level, PT, of a given protein, P, in an adhesion site is the sum of its levels in all its assemblies there. (c) The model was simulated with different noise and diversity levels, as described. For each possible pair of simulated noise and diversity levels, the signs of changes in these levels were inferred based on the Δr2 between the total levels of the indicated components and ΔCV of the first one. Red bars show the fraction of observations in each category. Blue stripes indicate the expected possible changes in noise and diversity based on the inference rules (Fig 2d). (PDF) [file pone.0160591.s005.pdf]

a

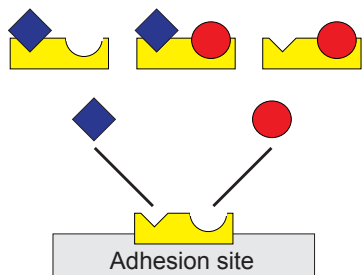

b

$$A^T = A + AB + AC + ABC$$

$$B^T = AB + ABC$$

$$C^T = AC + ABC$$

c

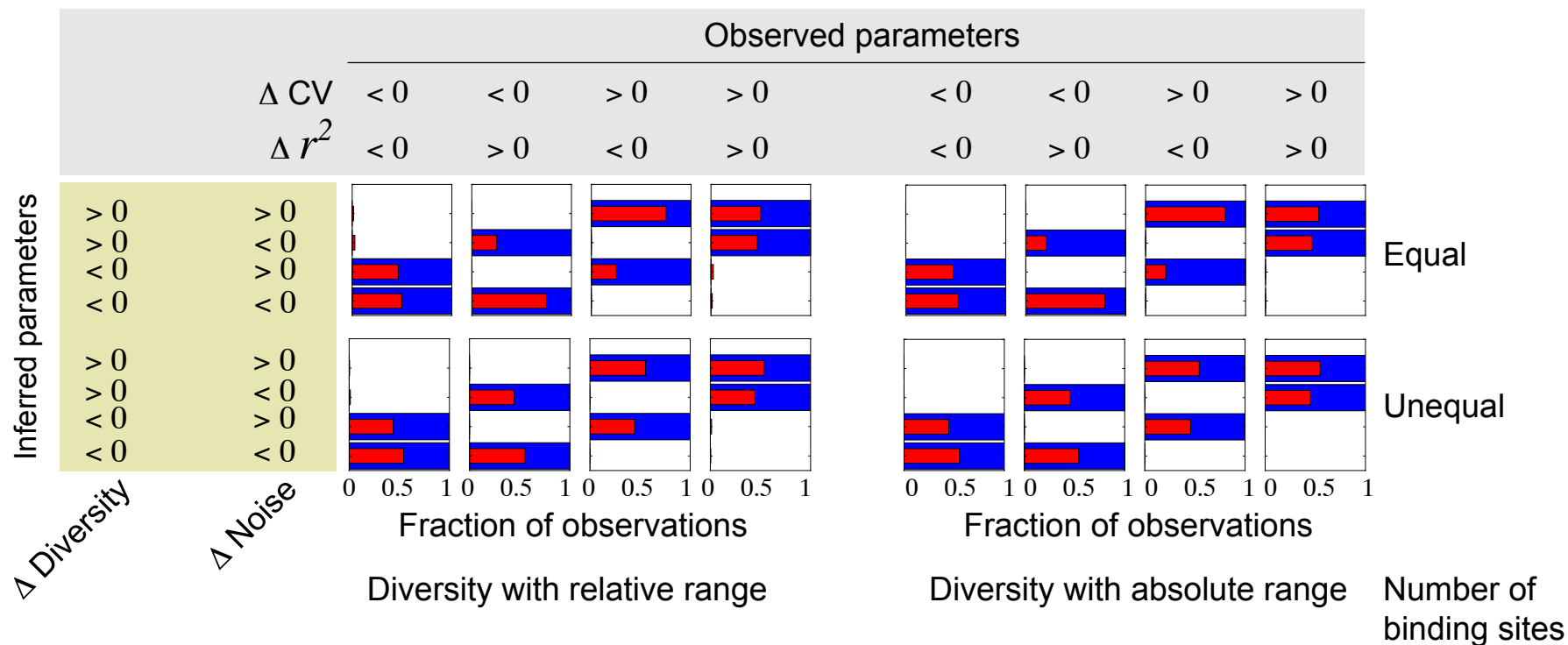

d

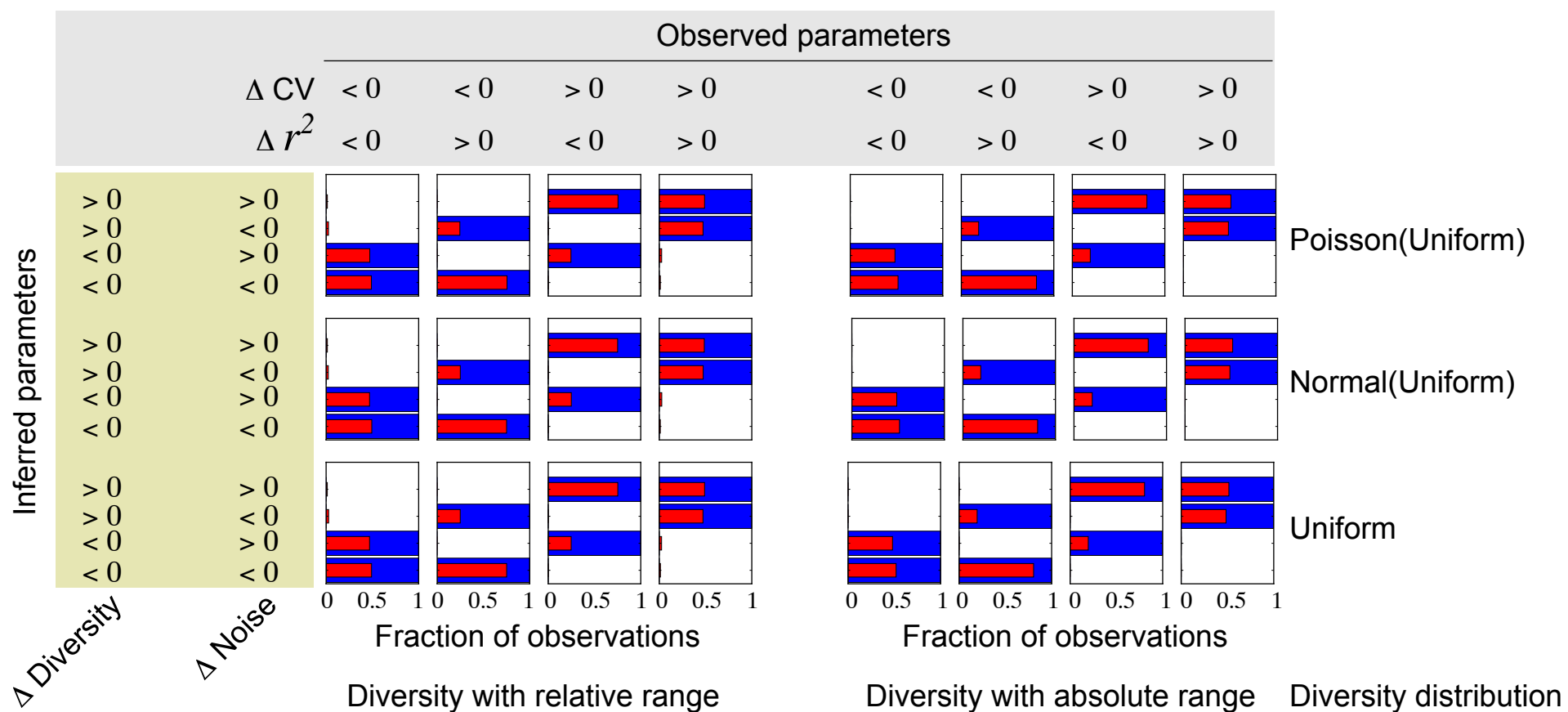

Supplement: S5 Fig — (a) The model and its letter notation. The level of component A is varying among sites due to diversity in local cues. (b) The total level, PT, of a given protein, P, in an adhesion site is the sum of its levels in all its assemblies there. (c) The model was simulated with different noise and diversity levels, diversity mean/range ratios and equal or unequal binding sites for proteins B and C, as described. For each possible pair of simulated noise and diversity levels, the signs of changes in these levels were inferred based on the Δr2 between AT and BT and ΔCV of AT. Red bars show the fraction of observations in each category. Blue stripes indicate the expected possible changes in noise and diversity based on the inference rules (Fig 2d). (d) As (c), using different diversity distributions as described. (PDF) [file pone.0160591.s006.pdf]

a

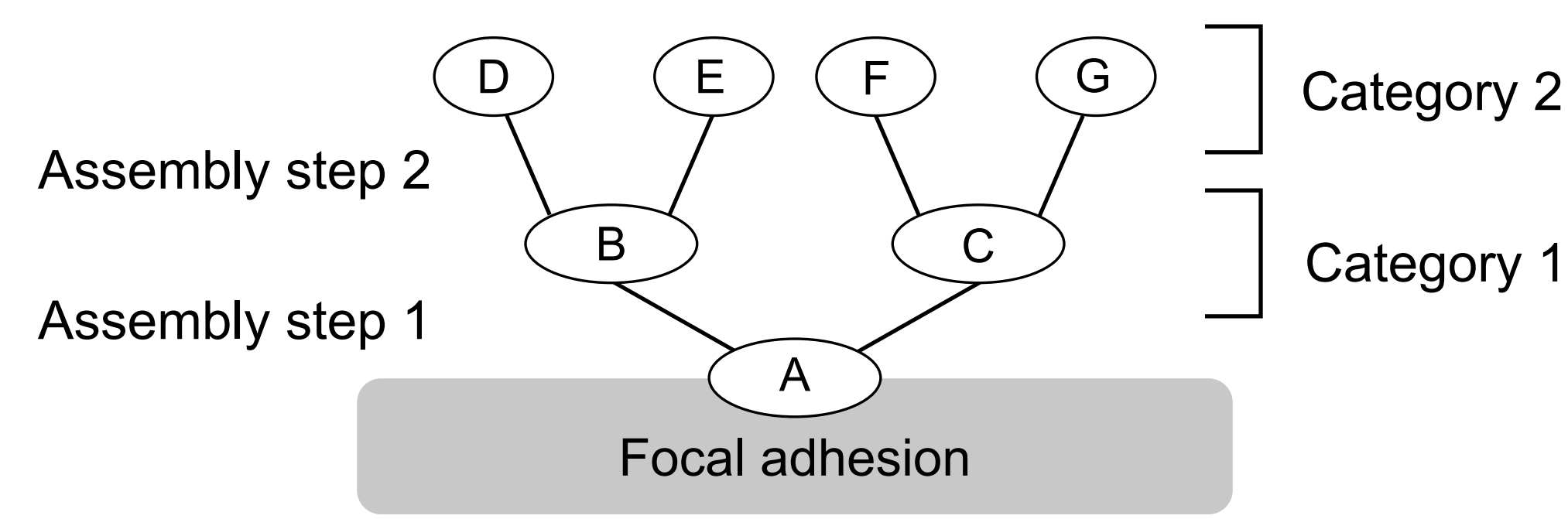

b

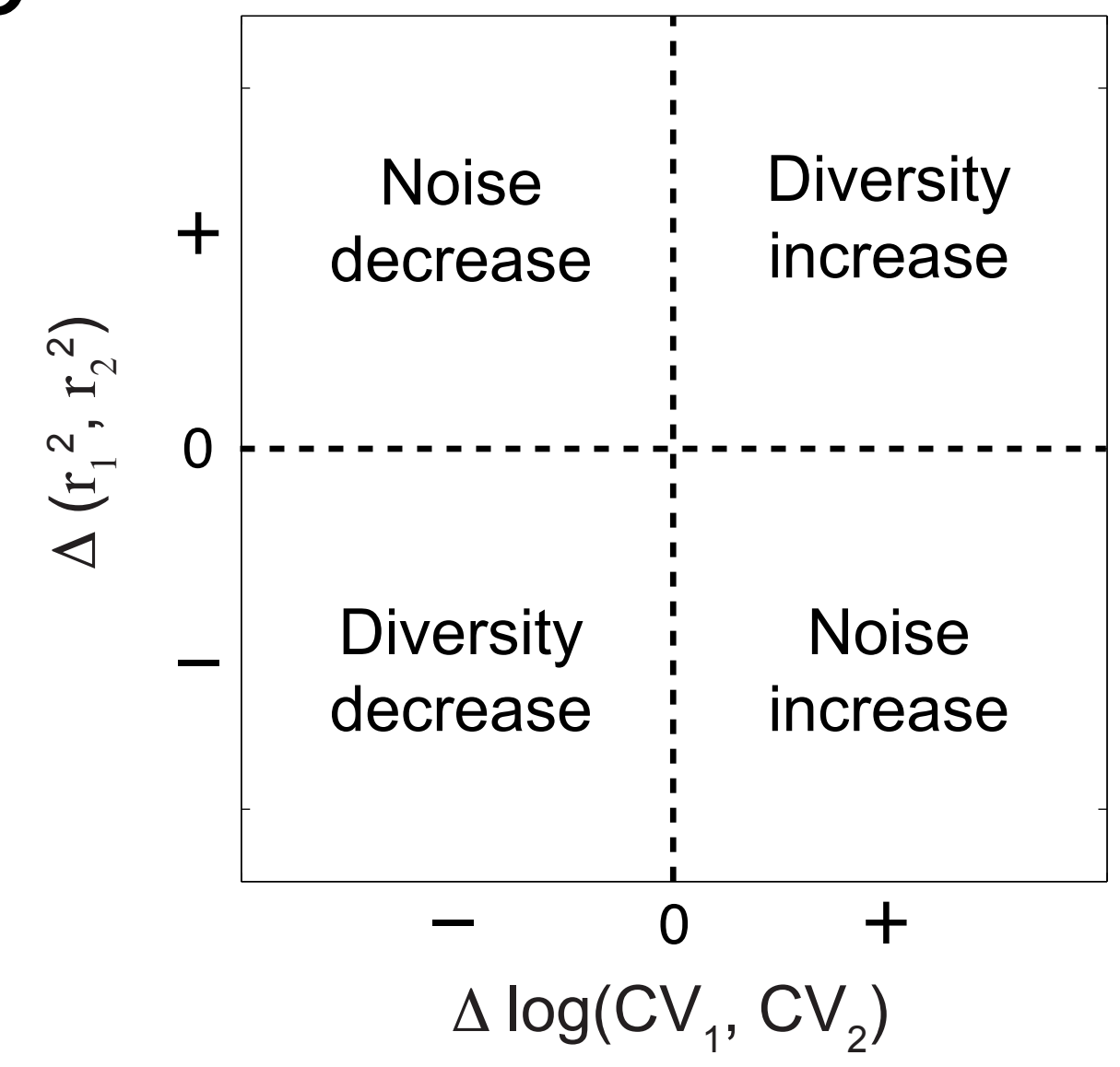

c

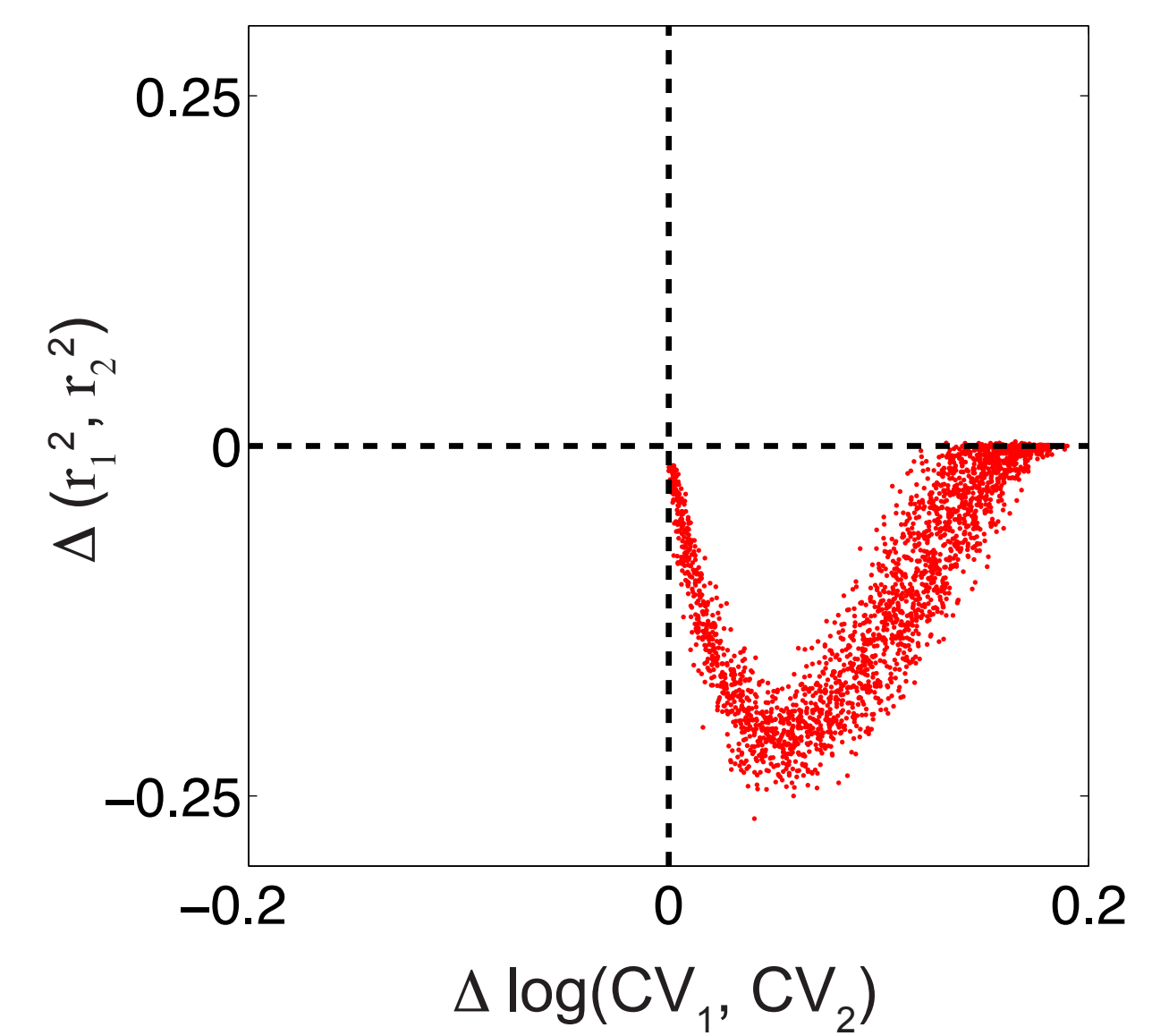

d

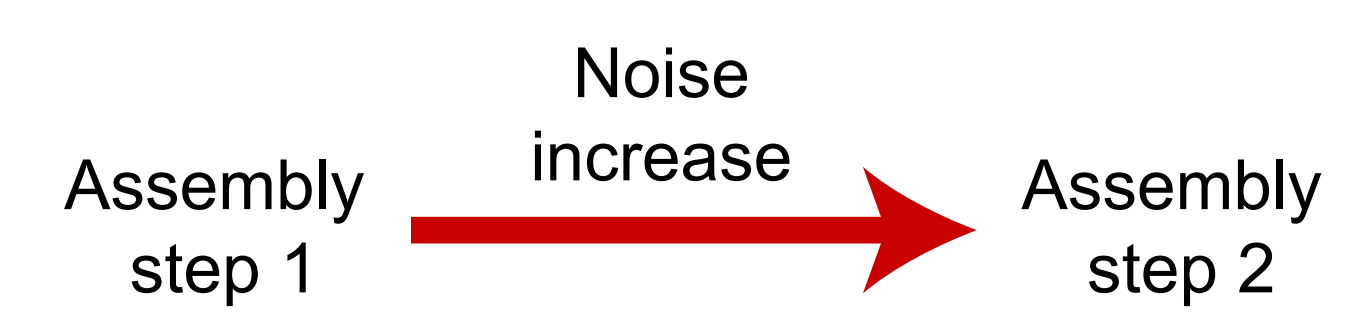

Supplement: S6 Fig — (a) The simulated model, consisting of two layers of non-competitive interactions. The level of component A is varying from site to site due to diversity in local cues. (b) The noise inference scheme. (c) Changes in Δlog(CV) and Δ(r2) between proteins recruited in the first assembly step (category 1) and those recruited in the second step (category 2). (d) Inferred changes in noise levels between the sequential assembly steps. (PDF) [file pone.0160591.s007.pdf]

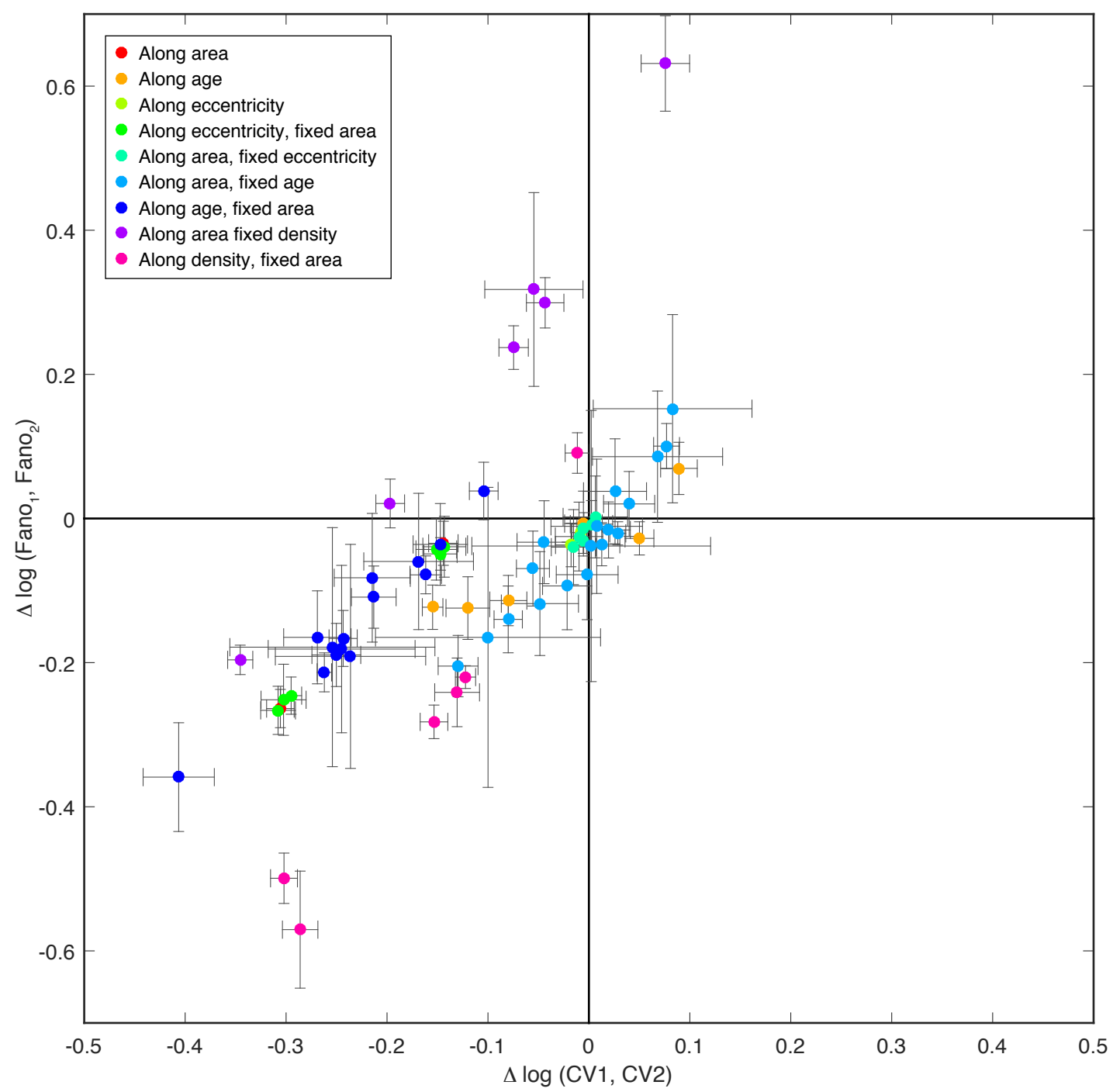

Supplement: S7 Fig — Note that beside few exceptions, the changes in these two measures have the same sign. Error bars indicate standard error of the mean between the datasets (see S1 Fig and S2 Table). (PDF) [file pone.0160591.s008.pdf]

**a**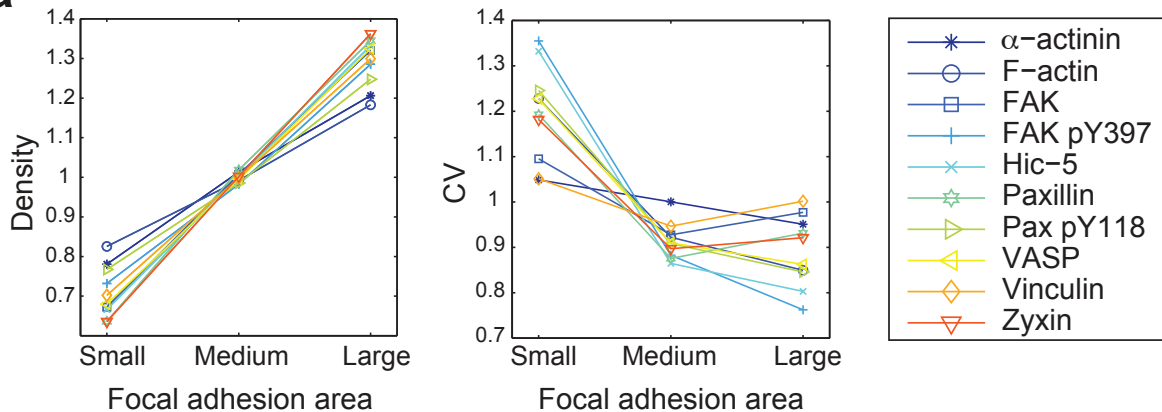**b**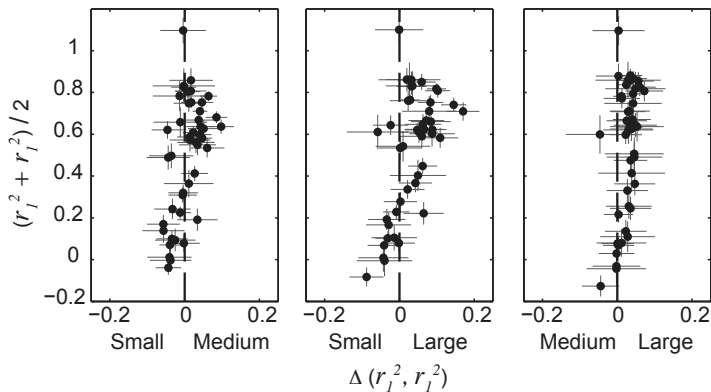**c**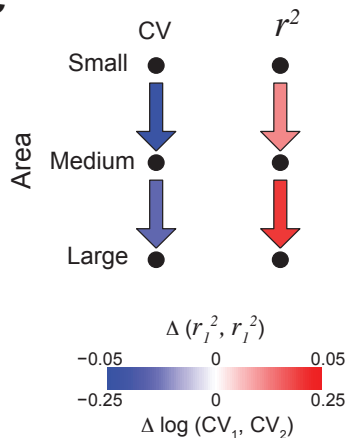

Supplement: S8 Fig — (a) The mean densities (n = 6 datasets) of the labeled components and their CV as a function of focal adhesions area. (b) Scatter plots comparing the r2 between the components in focal adhesions between the area categories. Error bars indicate standard error of the mean (n = 6 datasets). (c) Δlog(CV) and Δ(r2) as a function of focal adhesions area. (PDF) [file pone.0160591.s009.pdf]

**a**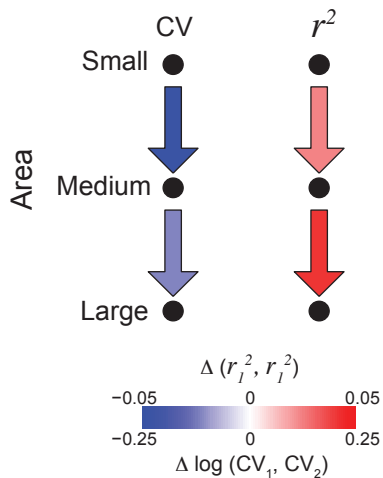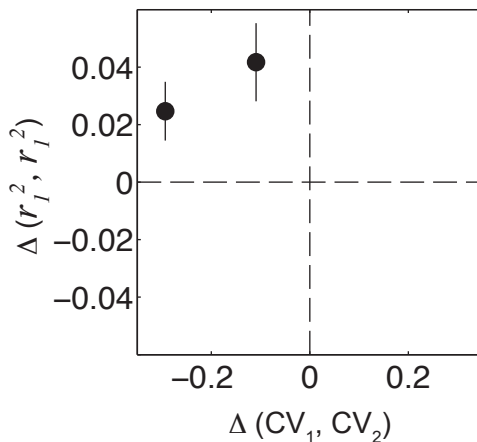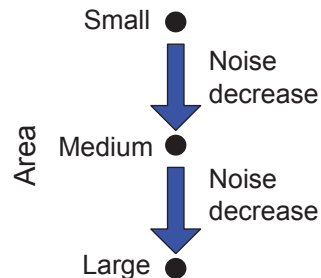**b**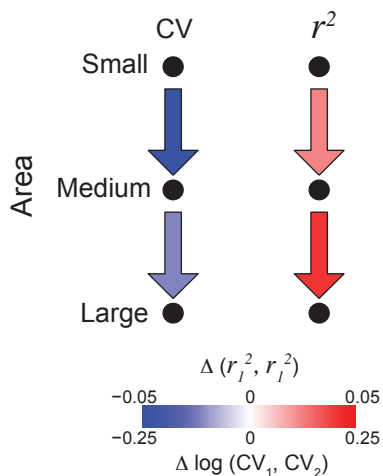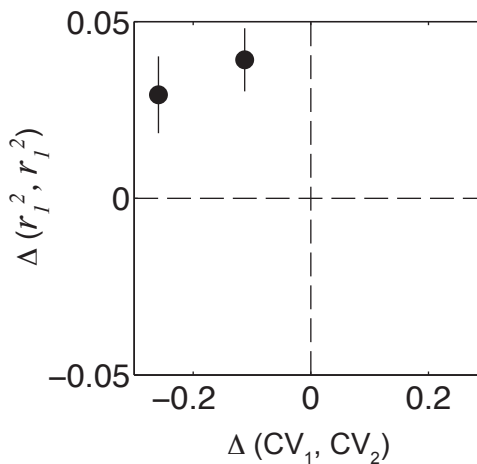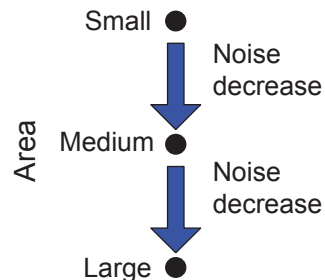

Supplement: S9 Fig — (a) Inferring changes in noise levels as a function of focal adhesions area based on Δlog(CV) and Δ(r2). However, here, the densities of proteins in each focal adhesion in the medium and large size categories were calculated using only a randomly sampled fraction of the pixels, such that the mean number of pixels sampled per focal adhesion is equal among all area categories. (b) The same as (a), but with sampling an equal number of pixels (10 pixels) from each focal adhesion in all area categories. Note that in both (a) and (b) the number of pixels used for calculating the CV and r2 is equal for all compared area categories, yet without affecting the detection of the reduction in the noise level. Error bars indicate standard error of the mean (n = 6 datasets). (PDF) [file pone.0160591.s010.pdf]

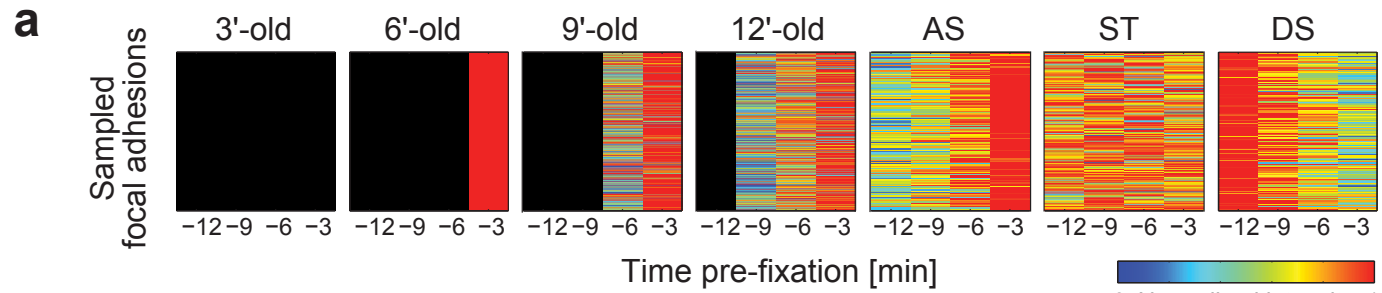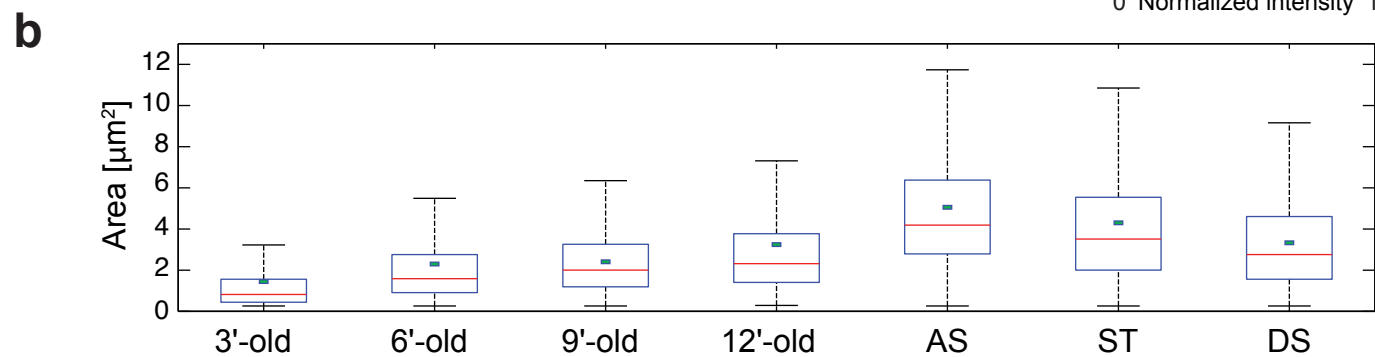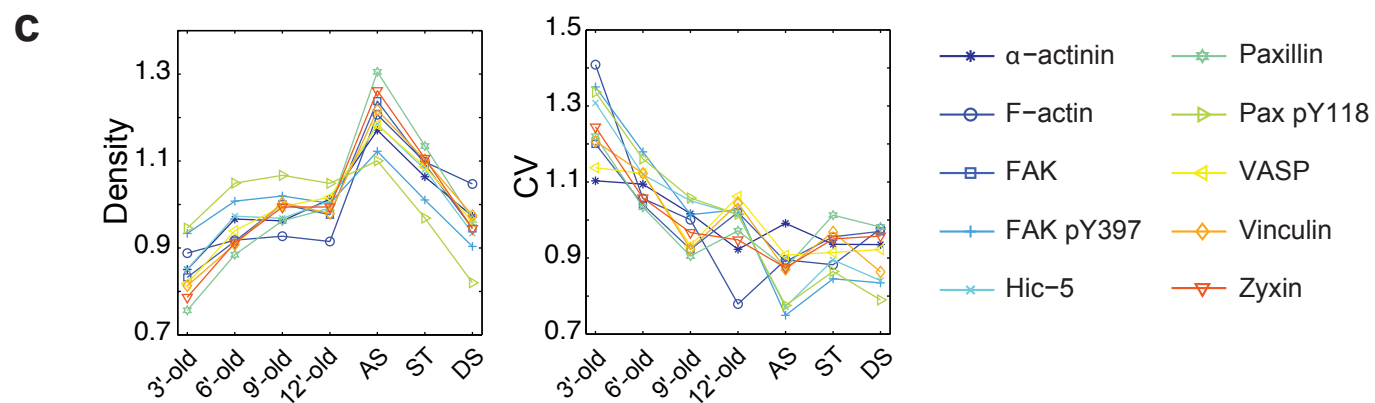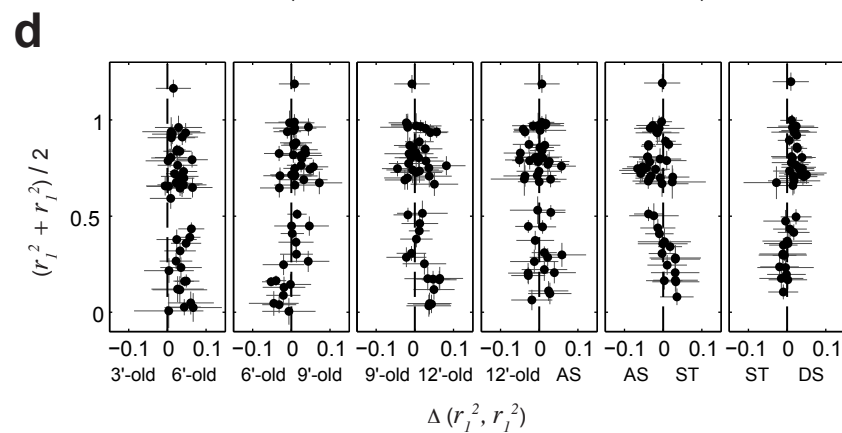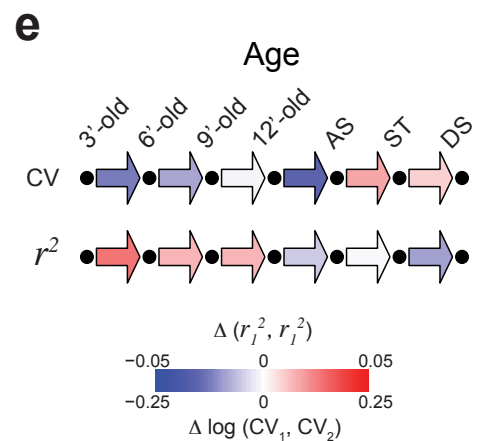

Supplement: S10 Fig — (a) The total intensity of YFP-paxillin during the last 12 minutes before fixation in individual, randomly sampled, focal adhesions of the different age categories (b) The areas of focal adhesions in each age category. Error bars denote standard deviation (n = 6 datasets). (c) The mean densities and CV (n = 6 datasets) of the labeled components as a function of focal adhesions age. (d) Scatter plots comparing r2 between the component densities in focal adhesions of sequential age categories. Error bars indicate standard error of the mean (n = 6 datasets). (e) Δlog(CV) and Δ(r2) as a function of focal adhesions age. (PDF) [file pone.0160591.s011.pdf]

**a**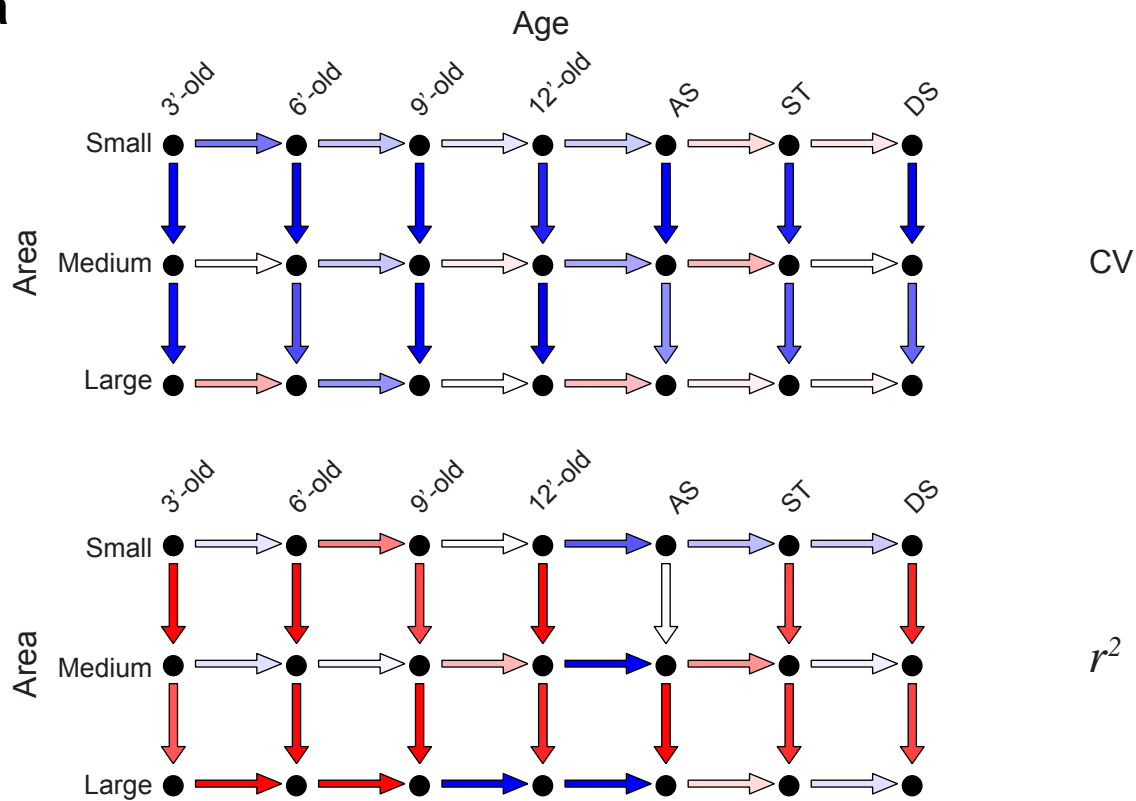**b**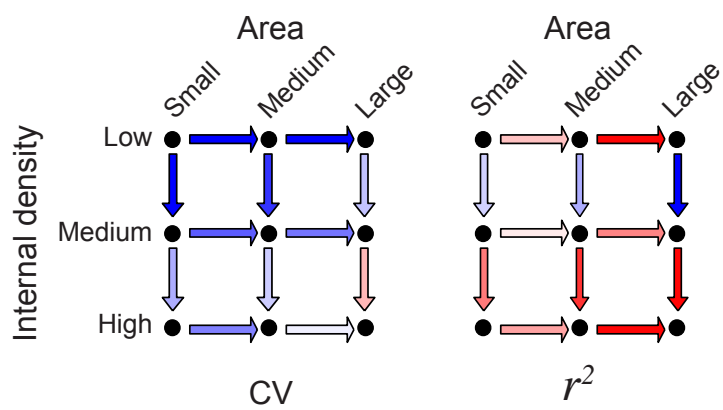**c**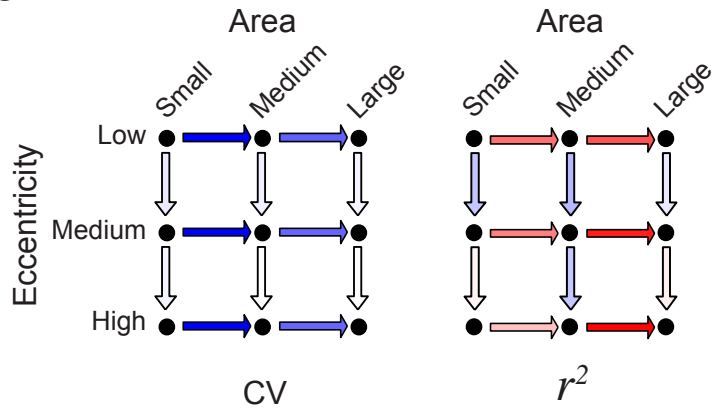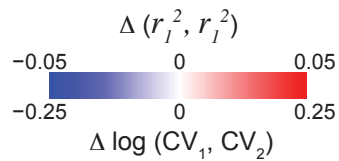

Supplement: S11 Fig — (a) Focal adhesions were sub-categorized according to both their area and age. Δlog(CV) and Δ(r2) were calculated between focal adhesions of the same age category as a function of area, as well as between focal adhesions of the same area category as a function of age. (b) As (a), using density instead of age. (c) As (a), using eccentricity instead of age. (PDF) [file pone.0160591.s012.pdf]

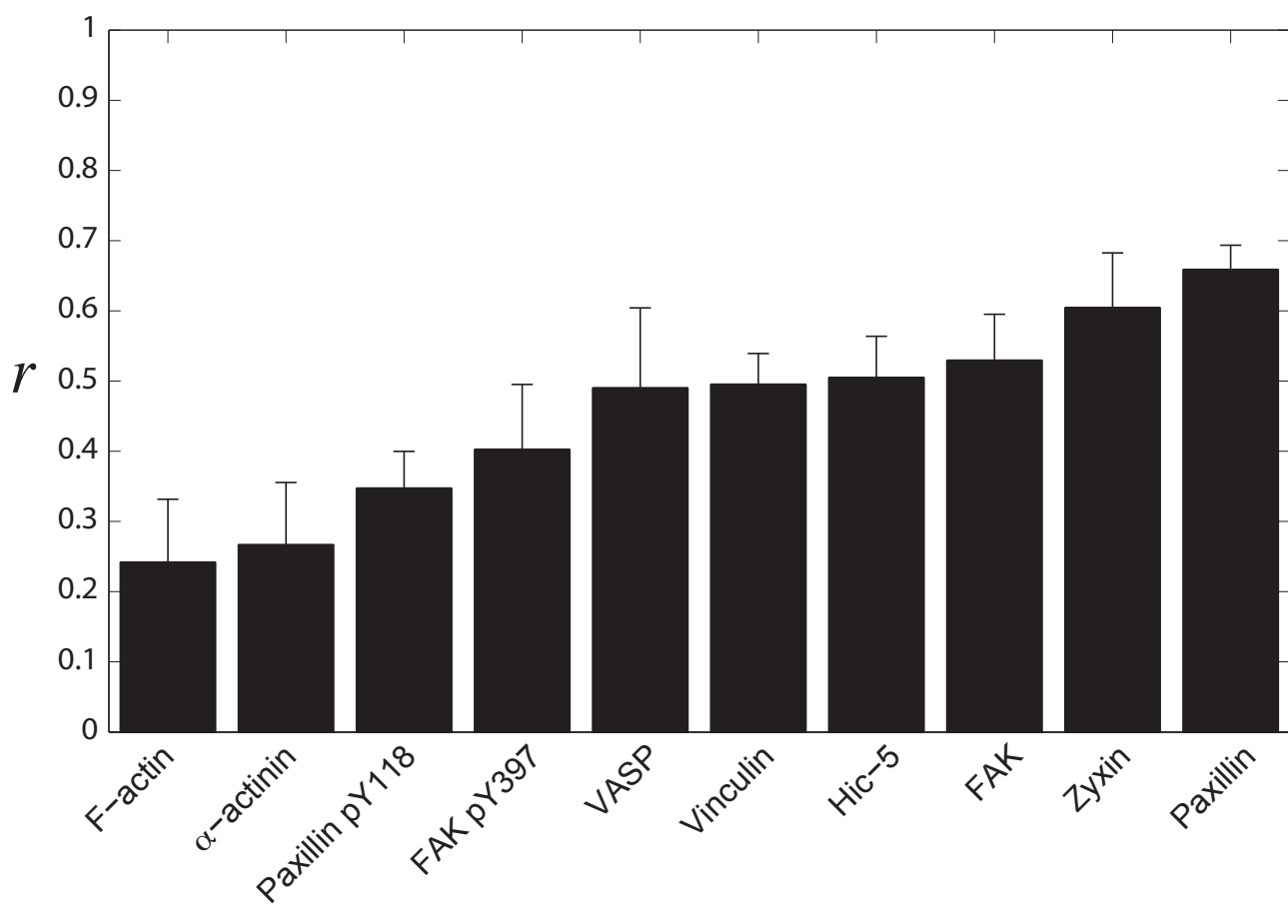

Supplement: S12 Fig — Error bars indicate standard error of the mean (n = 6 datasets). (PDF) [file pone.0160591.s013.pdf]

**a**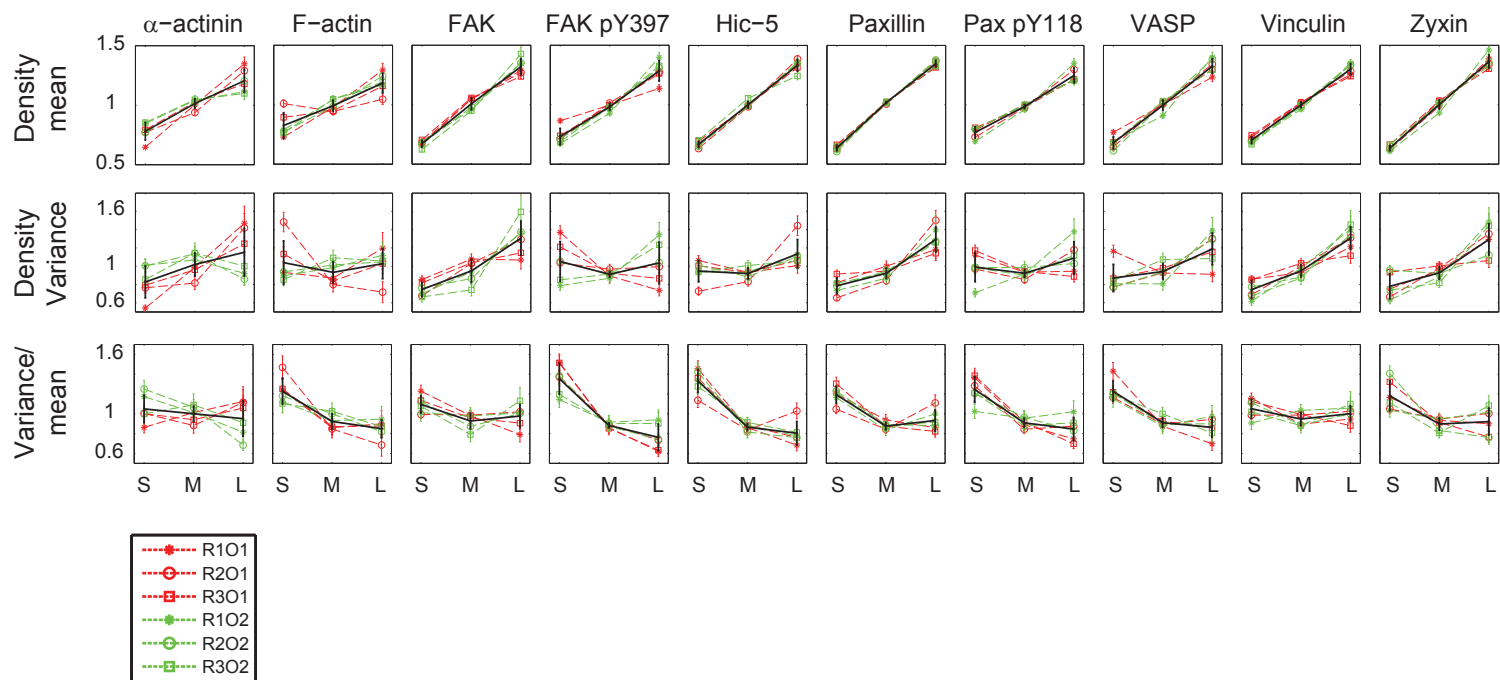**b**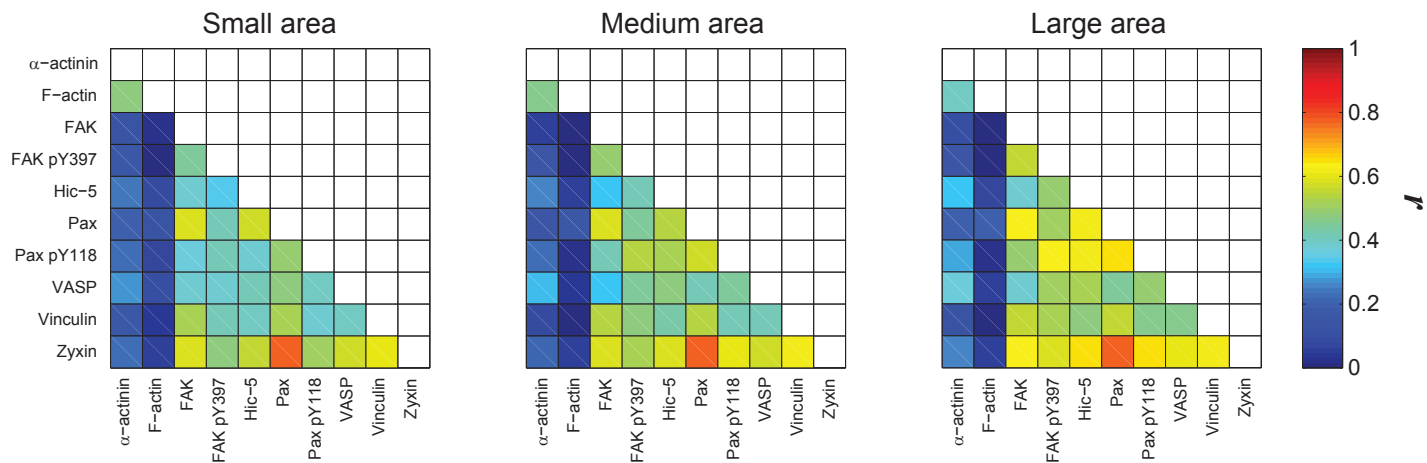

Supplement: S13 Fig — (a) The mean, variance and CV of the density of the various components in focal adhesions of the different area categories and in the different datasets. S, M and L denote the small, medium and large area categories, respectively. Error bars indicate standard error of the mean (see S1 Fig for the number of analyzed focal adhesions for each dataset and area category). (b) The mean Pearson correlation, r, (n = 6 datasets) between the densities of the components in focal adhesions of the different area categories. (PDF) [file pone.0160591.s014.pdf]

**a**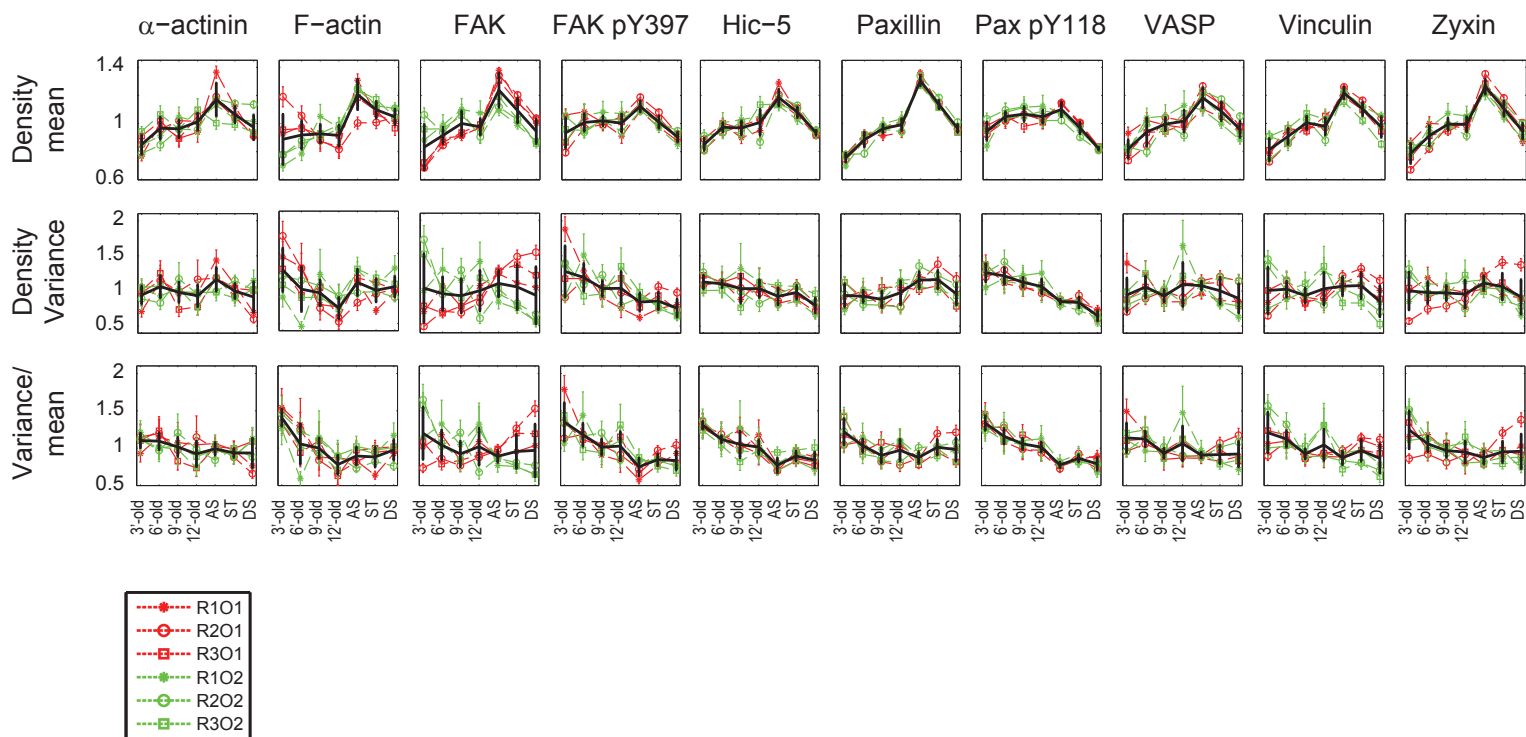**b**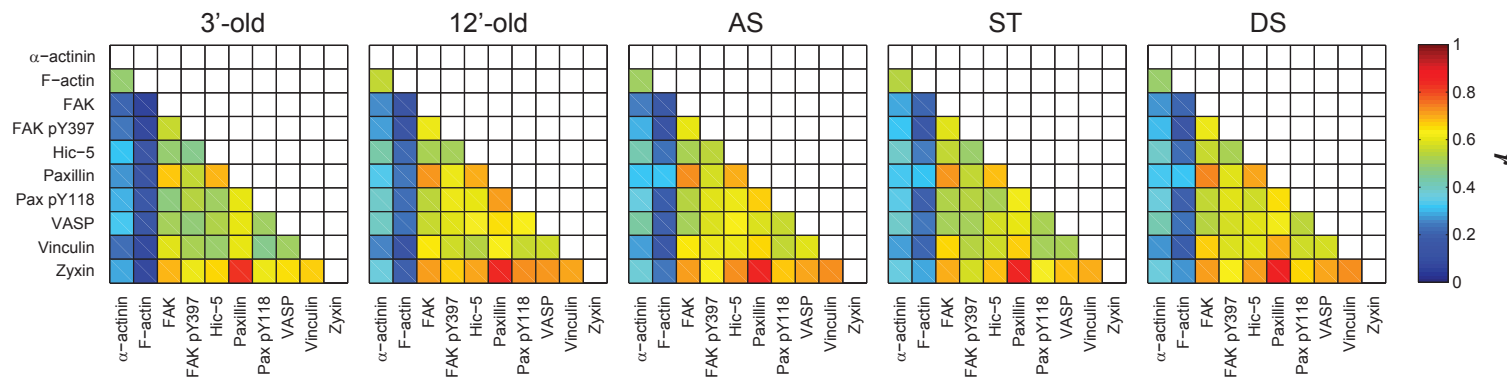

Supplement: S14 Fig — (a) The mean, variance and CV of the density of the various components in focal adhesions of the different age categories and in the different datasets. Error bars indicate standard error of the mean (see S1 Fig for the number of analyzed focal adhesions for each dataset and age category). (b) The mean Pearson correlation, r, (n = 6 datasets) between the densities of the components in focal adhesions of the different age categories. (PDF) [file pone.0160591.s015.pdf]
