# Supplementary material for: Highly Multiplexed Imaging Uncovers Changes in Compositional Noise within Assembling Focal Adhesions
Source: PLoS One. 2016 Aug 12;11(8):e0160591. doi: 10.1371/journal.pone.0160591 (PMC4982658; doi:10.1371/journal.pone.0160591)
Supplement: S1 Table — (PDF) [file pone.0160591.s016.pdf]

Supplementary Table S1

Order	Cycle 1	Cycle 2	Cycle 3	Cycle 4
1	Vinculin<br>FAK<br>FAK pY397	Zyxin<br>VASP	Paxillin<br>Paxillin pY118	F-actin<br>Hic-5<br>α-Actinin
2	F-actin<br>Hic-5<br>α-Actinin	Paxillin<br>Paxillin pY118	Zyxin<br>VASP	Vinculin<br>FAK<br>FAK pY397

Supplementary Table S1. The two labeling orders of the components in the CycIF imaging cycles.
